# New insights into the 17β-hydroxysteroid dehydrogenase type 10 and amyloid-β 42 derived cytotoxicity relevant to Alzheimer’s disease

**DOI:** 10.1186/s13195-025-01821-8

**Published:** 2025-07-23

**Authors:** Aneta Houfková, Monika Schmidt, Ondřej Benek, Ivo Fabrik, Rudolf Andrýs, Lucie Zemanová, Ondřej Soukup, Kamil Musílek

**Affiliations:** 1 Faculty of Science, Department of Chemistry, University of Hradec Kralove, Rokitanskeho 62, Hradec Kralove, 500 03 Czech Republic; 2 Biomedical Research Centre, University Hospital Hradec Kralove, Sokolska 581, Hradec Kralove, 500 03 Czech Republic

**Keywords:** 17β-hydroxysteroid dehydrogenase type 10, Amyloid-β peptide, Alzheimer’s disease, Amyloid precursor protein, Mitochondria

## Abstract

**Background:**

The mitochondrial enzyme 17β-hydroxysteroid dehydrogenase type 10 (HSD10) is implicated in neurodegenerative disorders, particularly Alzheimer’s disease (AD), through its interplay with the amyloid-β peptide (Aβ). However, its independent pathological role in AD remains unclear.

**Methods:**

To explore the individual effects of HSD10 and amyloid precursor protein (APP) overexpression (including the Aβ42-generating APP_Swe/Ind_ variant), monoclonal HEK293 cell lines were developed. Cellular fitness was evaluated by measuring ATP levels, cell viability, and cytotoxicity measurements under glucose and galactose culture conditions. Mitochondrial metabolic changes were analysed using mitochondrial electron flow measurements in response to various metabolic substrates. HSD10 enzymatic activity was monitored using a fluorogenic probe, and two HSD10 inhibitors were tested for their ability to reduce cytotoxic effects. Statistical significance was determined using appropriate tests as detailed in the methods section.

**Results:**

The overexpression of HSD10 or APP_Swe/Ind_ led to mitochondrial dysfunction and reduced viability, particularly under glucose-deprived conditions. HSD10-driven cytotoxicity was linked to its enzymatic activity and associated with impaired TCA cycle function, reduced β-oxidation, and increased oxidative stress. In contrast, APP_Swe/Ind_ overexpression induced Aβ42 production, glucose hypermetabolism, and enhanced β-oxidation. Aβ42 also affected HSD10 activity and further amplified its cytotoxic effects. The benzothiazole-based HSD10 inhibitor **34** restored cell viability under both HSD10 overexpression and Aβ42-rich conditions.

**Conclusions:**

HSD10 and Aβ42 each contribute to mitochondrial impairment via distinct metabolic pathways. These findings established HSD10 as an independent pathological factor in AD and support the potential of HSD10 inhibitors, particularly inhibitor **34**, as therapeutic agents targeting mitochondrial dysfunction in AD.

**Supplementary Information:**

The online version contains supplementary material available at 10.1186/s13195-025-01821-8.

## Background

17β-hydroxysteroid dehydrogenase type 10 (HSD10, UniProt: Q99714), a member of the short-chain dehydrogenase/reductase superfamily, is a multifunctional mitochondrial NAD^+^-dependent enzyme involved in fatty acid metabolism, isoleucine degradation, and steroid metabolism [1]. Besides its enzymatic activity, HSD10 is also involved in mitochondrial tRNA maturation and its further downstream processing [2–4]. HSD10 protein was found essential for normal mitochondrial function and neuronal development, as mutations in the HSD10 gene impacting the protein´s structural stability have been associated with a rare X chromosome-linked inborn error of metabolism which causes rapid and progressive neurodegeneration prevalent in neonatal and infant age [5–7]. The changes in HSD10 levels and/or its enzymatic function are connected with several diseases, particularly with neurodegenerative disorders such as Alzheimer’s disease (AD) [1] and Parkinson’s disease [8]. In AD, elevated levels of HSD10 were found in affected brain regions, where HSD10 is considered to mediate amyloid-β peptide (Aβ)-associated cytotoxicity, and this interplay is thought to be one of the possible aspects of AD pathogenesis [9, 10].

In AD progression, the engagement of HSD10 and its interplay with Aβ results in mitochondrial dysfunction and neuronal destruction [9–11]. Aβ peptide is the cleavage product of amyloid precursor protein (APP), derived via APP’s subsequent amyloidogenic splicing by β- and γ-secretases. Aβ and its deposits are one of the histopathological hallmarks found in AD patients’ brains. Aβ produced by APP cleavage can range from 36 to 43 amino acids [12]. Aβ peptide is a normal metabolic product present even in healthy individuals, generated through the physiological cleavage of APP. However, excessive accumulation or altered processing of APP leading to abnormal Aβ species is associated with AD pathology and neurotoxicity [13]. In relation to human AD brain deposits, the Aβ42 long fragment dominates, and its oligomeric forms are considered neurotoxic Aβ species [14, 15].

Importantly, APP is not only a pathological precursor of Aβ but also a vital protein with essential physiological functions in the brain. Full-length APP acts as a receptor involved in signal transduction via G-protein interactions [16] and contributes to cell adhesion by binding various extracellular molecules or facilitating intercellular contact [17]. The binding of specific ligands, such as Aβ, F-spondin, or netrin-1, to the extracellular domain of APP can further modulate its processing and downstream signaling pathways [18–20]. Additionally, studies using APP gene silencing have revealed its role in regulating neuronal migration during early brain development [21, 22]. These physiological functions are particularly important during neurodevelopment but also support synaptic plasticity and cognitive performance in the adult brain.

**Fig. 1:**
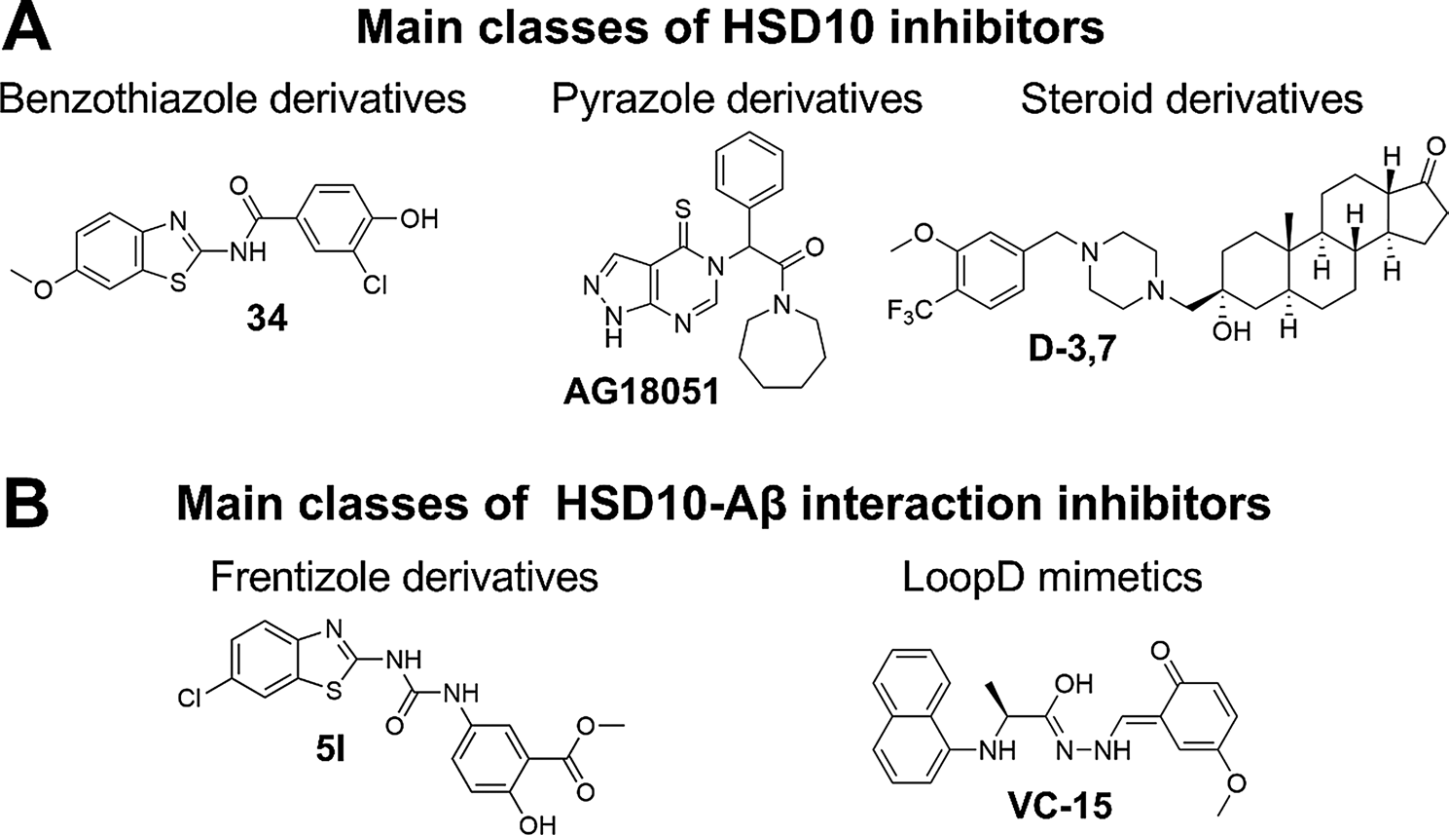
(**A**) Representatives of the three main classes of HSD10 inhibitors– benzothiazole derivatives (**34**) [37], pyrazole derivatives (**AG18051**) [38], and steroid derivatives (**D-3**,**7**) [36]. (**B**) Representatives of two main classes of inhibitors of HSD10-Aβ interaction– frentizole derivatives (**5l**) [33] and loopD mimetics (**VC-15**) [34]

The mitochondrial dysfunction, as a consequence of HSD10 and Aβ peptide interplay, is characterized by elevated oxidative stress, increased reactive oxygen species (ROS) production, inhibition of mitochondrial complex IV, altered ATP (adenosine triphosphate) level, the release of lactate dehydrogenase and cytochrome c from the mitochondria, DNA fragmentation, and increased apoptosis, and results in cell death [9–11, 23–25]. According to the available publications, HSD10 is thought to mediate Aβ-driven cytotoxicity, probably in a manner where Aβ affects HSD10 enzymatic function [24–26]. The HSD10-Aβ interaction was studied using recombinant HSD10 enzyme, resulting in the determination of a dissociation constant of 55.8 nM for Aβ42 [10]. Based on the literature, preventing the HSD10-Aβ interplay and/or modulation of HSD10 enzymatic function offers potential opportunities for drug development [27–29]. To date, several classes of HSD10 inhibitors or inhibitors of HSD10-Aβ interaction have been published (Fig. 1) [27, 30–36].

However, further development and evaluation of the aforementioned HSD10 inhibitors require a better understanding of their potential to mitigate the HSD10 overexpression-related pathology. Thus, without a thorough understanding of the pathological role of HSD10 overexpression in AD, it is difficult to accurately assess and compare HSD10 inhibitors and their potential as effective therapies. This study hence aimed to describe the effects of individual HSD10 and APP overexpression on cellular fitness, with the main focus on HSD10’s role in cytotoxicity development, both independently and in the presence of Aβ. In addition, the acquired findings were used to develop a novel model for testing HSD10 inhibitors, enabling the scoring of their ability to reduce pathology linked to HSD10 and Aβ, two critical factors in AD progression.

## Materials and methods

### Chemicals and compounds

The majority of the chemicals used were obtained from Sigma-Aldrich unless otherwise noted. All chemicals were of the highest commercially available purity. The tested HSD10 inhibitors were chosen from the in-house library of the University of Hradec Kralove, Faculty of Science. The synthetic routes and chemical properties can be found in the published articles [38–40]. All compounds were dissolved in anhydrous dimethyl sulfoxide (DMSO) and further diluted with assay buffers or culture media to the working concentrations to keep maximum DMSO concentration at 2% (v/v) for HSD10 activity measurements and at 1% (v/v) for other cell culture experiments.

### Cell cultures and HSD10 overexpression or APP_Swe/Ind_ overexpression

HEK293 cells (ECACC Cat# 85120602, RRID: CVCL_0045) were commonly cultured in Dulbecco´s modified Eagle medium (Capricorn; DMEM) supplemented with 10% fetal bovine serum (Gibco), 2 mM L-glutamine (Gibco), and non-essential amino acid additives (Gibco) at 37 °C in a 6% CO_2_ humidified atmosphere (standard DMEM medium with phenol red). The cells were passaged regularly at 70–80% confluency and routinely tested for *Mycoplasma* contamination (MycoAlert Plus, Promega). Cell lines have been authenticated during the preparation of the manuscript [31]. All experiments were performed at a maximum passage number of 15 (the passage number counting started at the time of purchase). Glucose (4.5 g/L) or galactose (1.8 g/L) Dulbecco’s modified Eagle medium (Gibco, A1443001) without the addition of phenol red supplemented with 0.11 g/L sodium pyruvate, 10% fetal bovine serum (Gibco), 2 mM L-glutamine and non-essential amino acid additives (Gibco) were used for all cell culture experiments. Expi293 Expression Medium (Gibco, A1435101) was used for Western blot analysis, size-exclusion chromatography, and mass-spectrometry analysis of the conditioned culture medium.

For APP_Swe/Ind_ overexpression, pCAX APP695 Swe/Ind vector (pCAX APP Swe/Ind was a gift from Dennis Selkoe [21] & Tracy Young-Pearse (Addgene plasmid # 30145; http://n2t.net/addgene:30145; RRID: Addgene_30145)) was used as a template. The full-length gene for APP_Swe/Ind_ was PCR amplified and inserted into a constitutive mammalian expression pcDNA3.4 vector using topoisomerase-based cloning, and the insertion was subsequently sequenced. Low-passaged wild-type HEK293 cells (HEK293_wt_; human embryonic kidney cells) were nucleofected (Amaxa Nucleofector kit V, Lonza, nucleofection program Q-001) with 3 µg of pcDNA3.4-APP_Swe/Ind_ vector plasmid DNA isolated using PureLink HiPure Plasmid MiniPrep kit (Invitrogen). Twenty-four hours after nucleofection, the cells were treated with G418 disulfate salt solution (Roche) at a concentration of 500 µg/mL in culture medium to select positive cell clones. After several weeks of clonal selection and expansion, several monoclonal cell lines (referred to as APP_Swe/Ind_ cells) were isolated using the limiting dilution method. The APP_Swe/Ind_ overexpression and subsequent Aβ42 overproduction were confirmed by immunoblotting using anti-APP and anti-Aβ primary rabbit monoclonal antibodies (Table 1). For HSD10 overexpression, HSD10 overexpressing cells published previously were used [31]. For the preparation of HSD10 overexpression cell lines harbouring the inactivated catalytic active site of the enzyme (Y168A, K172A) (referred to as HSD10_mut_ cells), the HSD10 DNA was modified accordingly, prepared *de novo* as a DNA string (GeneArt, ThermoFisher), and cloned into the pcDNA3.4 vector. After DNA sequencing verification, the same procedure was followed as for APP_Swe/Ind_ cell line preparation. The final confirmation of the expression level was performed using an anti-ERAB monoclonal antibody (Table 1). No institutional ethical approval was required for this work.

### Western blot analysis

The expression levels of particular proteins in the overexpressed cell lines were confirmed using sodium dodecyl sulfate-polyacrylamide gel electrophoresis (SDS-PAGE) 10% (APP_Swe/Ind_ detection) or 15% (HSD10, HSD10_mut,_ and Aβ detection) resolving gel and 4% stacking gel. The protein samples under reducing conditions were subjected to electrophoresis (10 µg/20 µg of whole cell lysates from HEK293_wt_, HSD10, HSD10_mut,_ or APP_Swe/Ind_ cell lines or 14 µL of conditioned culture media from HEK293_wt_ or APP_Swe/Ind_ lines), subsequently transferred to 0.45 μm PVDF membrane, and detected using a primary antibody followed by chemiluminescence detection of HRP-conjugated secondary antibody (Amersham ECL Prime). β-actin as a loading control was used for analysis of whole cell lysates. The gel staining was used as a loading control for the conditioned medium analysis.

Primary and secondary antibodies are listed in Table 1.

**Table 1:** Primary and secondary antibodies used for Western blot analysis

Detected protein	Primary Antibody	Secondary Antibody
HSD10	Anti-ERAB Antibody, Rabbit mAb, Abcam, Cat# ab167410, dilution 1:50000	Goat Anti-Rabbit IgG (H + L) Secondary Antibody, Invitrogen, Cat# 31,460, dilution 1:5000
APP_Swe/Ind_	Anti-Amyloid Precursor Protein Antibody, Rabbit mAB, Abcam, Cat# ab32136, dilution 1:1000	
Aβ	Anti-β-Amyloid Antibody (D54D2), Rabbit mAb, Cell Signaling, Cat# 8243, dilution 1:1000	
	Anti-β-Amyloid (1–40) Antibody, Mouse mAb, Invitrogen, Cat# LF-MA0152, dilution 1:2000	Goat Anti-Mouse IgG (H + L) Secondary Antibody, Invitrogen, Cat# 32,430, dilution 1:1000
β-Actin	Anti-β-Actin Antibody, Mouse mAB, ABclonal Technology, Cat# AsC004, dilution 1:20000	Goat Anti-Mouse IgG (H + L) Secondary Antibody, Invitrogen, Cat# 32,430, dilution 1:1000

### ATP level and cytotoxicity determination

The impact of overexpression of particular proteins (HSD10, HSD10_mut,_ or APP_Swe/Ind_) or HSD10 inhibitors on ATP production and the potential cytotoxic effects was tested using CellTiter-Glo Luminescent Cell Viability Assay and CellTox Green Cytotoxicity Assay kits (Promega, G7574 and G8741), respectively. For multiplex measurement, 5 × 10^3^ cells were seeded in 50 µL of glucose or galactose culture medium per well into a white 96-well microplate with a clear flat bottom (Corning, CLS3903). Cells were cultured for 24, 72, or 168 hr depending on the experiment setup. The assay readouts were measured using the TECAN Spark 10 M instrument following the manufacturer’s protocol with minor changes. For cytotoxicity determination, 20 µL of complete CellTox Green Reagent (mixing 20 µL of CellTox Green Dye with 6 mL of CellTox Green Assay Buffer) was used in combination with fluorescence readout (Ex/Em = 485/530 nm). For ATP production measurements, 25 µL of CellTiter-Glo 2.0 Reagent was used, followed by the luminescence-based measurement with an integration time of 500 ms. HEK293_wt_ cells treated with 1% DMSO only (vehicle control) and 100 µM valinomycin-treated cells (positive control) were used as the controls.

### Viability determination

The protein overexpression effect on cell viability was measured using RealTime-Glo MT Cell Viability Assay (Promega, G9711). The cells (5 × 10^3^) were seeded in 50 µL of complete glucose medium per well into a white 96-well microplate with a clear flat bottom (Corning, CLS3903). Cells were cultured for 24 hr to allow them to adhere, followed by changing the culture medium to galactose, immediately treating with 50 µL of galactose medium supplemented with MT Cell Viability Substrate and NanoLuc Enzyme in a final 2X concentration. The treated cells were incubated at 37˚C and 6% CO_2_ for 30 min, followed by 72 hr of luminescence-based viability monitoring (every 6–12 h) using a Tecan Spark 10 M instrument.

### Mitochondrial toxicity determination

A Mitochondrial ToxGlo Assay kit (Promega, G8000) was used to determine the effect of protein overexpression (HSD10 or APP_Swe/Ind_) on mitochondrial fitness. For measurement, 5 × 10^3^ cells were seeded in 50 µL complete galactose medium per well into a white 96-well microplate with a clear flat bottom (Corning, CLS3903), followed by 72 hr incubation time. Several control treatments were performed by adding 50 µL of control-containing media for different time intervals: positive toxicity treatment (100 µM valinomycin treated HEK293_wt_ cells) for 24 h; positive mitochondrial toxicity treatment (1 µM antimycin A treated HEK293_wt_ cells) for 90 min; and vehicle control (1% DMSO treated HEK293_wt_ or overexpressing cells) for 90 min. The mitochondrial dysfunction was determined as the activity of dead-cell proteases in combination with ATP level measurement. Complete Cytotoxicity Reagent treatment (20 µL; mixing of 10 µL bis-AAF-R110 substrate with 2 mL Mitochondrial ToxGlo Assay Buffer) was added to the cells for 30 min and measured fluorometrically (Ex/Em = 485/530 nm, Tecan Spark 10 M), followed immediately by treatment with 100 µL 2x ATP Detection reagent and luminescence measurement of ATP levels with an integration time of 500 msec.

### Preparation of Aβ42-containing medium

To generate a conditioned medium containing cell-derived Aβ42, APP_Swe/Ind_ cells were cultured in standard DMEM until reaching approximately 40% confluence. Before medium exchange, the cells were gently washed with phosphate-buffered saline (PBS) to remove residual phenol red. The growth medium was then replaced with either glucose-containing DMEM medium, galactose-containing DMEM medium, or EXPI293 medium, depending on the experimental condition. The cells were subsequently maintained in a fresh medium for 3–5 days to allow accumulation of secreted Aβ42.

After this incubation period, the conditioned medium was collected and centrifuged at 10,000 RCF for 10 min to remove cellular debris. The concentration of Aβ42 in the cleared supernatant was determined using the Amyloid beta 1–42 Kit (CisBio, 62B42PEG) based on homogeneous time-resolved fluorescence (HTRF) detection. The medium was then diluted to the desired Aβ42 concentration using freshly prepared, non-conditioned DMEM of the same composition (glucose or galactose) or EXPI293. The diluted conditioned medium was used immediately for cell treatment experiments without storage.

### Aβ42 quantification

To quantify the amount of Aβ42 secreted by the APP_Swe/Ind_ cell line, the Amyloid beta 1–42 Kit (CisBio, 62B42PEG) was used. The reaction mixture consisted of an HTRF binding pair of donor and acceptor antibodies (the Human Amyloid β 1–42 Europium cryptate antibody, and the Human Amyloid β 1–42 d2 antibody diluted with Detection buffer to 1X concentration, 2 µL each) and 16 µL of diluted sample medium (20 to 50X) or Aβ42 standard. The assay mixture was pipetted into an HTRF 96-well low-volume white plate (Revvity, 66PL96005), sealed, and incubated overnight at RT. Two sequential measurements of the FRET signal at 620 nm for Eu-cryptate emission (donor) and 665 nm for the d2 emission (acceptor) were measured, followed by calculation of the 665/620 ratio that was used for concentration determination based on the standard curve approximation.

### Cellular HSD10 Inhibition

HSD10 activity in the cellular environment was performed using (-)-CHANA (cyclohexenyl amino naphthalene alcohol) fluorogenic probe [31, 32, 41]. The cells were seeded at a density of 1 × 10^4^ cells per well in 200 µL of complete glucose medium into a black 96-well microplate with a clear bottom (Brand, BR781971). After 20 hr of incubation, the cells were treated with DMSO only, Aβ42-containing medium, or HSD10 inhibitors dissolved in anhydrous DMSO. After 2 hr of treatment, 2 µL of (-)-CHANA probe was added at the final concentration of 20 µM. Changes in fluorescent intensities were measured immediately (0 hr) and after 2 hr of incubation. The fluorescence intensities of the reaction product cyclohexenyl amino naphthalene ketone (CHANK) were measured using the TECAN Spark 10 M instrument (Ex/Em = 380/525nm). The HSD10 activity was calculated as the ΔF between 2 hr and 0 hr after (-)-CHANA addition, and the data were normalized between DMSO-treated HSD10 cells and non-transfected HEK293_wt_ controls (using relative response ratio).

### Cell metabolic activity changes

To investigate the changes in the cell metabolic activities caused by HSD10 or APP_Swe/Ind_ overexpression, the MitoPlate S-1 technology (Biolog) was used. The 96-well plates preloaded with 31 different substrates (tricarboxylic acid cycle (TCA) cycle intermediates, amino acids, fatty acids, hexoses, and trioses, each in triplicate) were activated by adding 30 µL of Assay Mix (15 µl 2x Biolog Mitochondrial Assay Solution; 10 µL 6x Redox Dye MC; 2.5 µL 24x saponin (Sigma, #47036) at 960 µg/mL, final concentration 40 µg/mL; and 2.5 µL sterile water) per well and incubated for 1 hr at 37 °C and 6% CO_2_. The cells were cultivated for 48 hr before the experiment in glucose or galactose complete media and then plated into the activated MitoPlate S-1 plates at a density of 3 × 10^4^ per well in 30 µL of 1X Biolog Mitochondrial Assay Solution. The reduction of the Redox Dye mix, which corresponds to changes in mitochondrial electron flow, was read by absorbance (OD_590_) kinetically on a Tecan Spark 10 M instrument at 30-sec intervals for a total of 4 hr after seeding. The data was extracted as ΔAbs per hour from the linear phase of the particular substrate consumption rates.

### Liquid chromatography-mass spectrometry analysis

APP_Swe/Ind_ conditioned cell culture medium was loaded to DSC-18 500 mg/mL SPE (Sigma), and bound material was eluted with 20% acetonitrile (ACN) and lyophilized. The lyophilizate was dissolved in reducing LDS buffer (Invitrogen), heated at 70 °C, divided into four lanes of NuPAGE precast gel (Invitrogen), and run by SDS-PAGE. The gel was stained, and visible bands were excised, chopped, and combined from each lane. Protein digestion was done as previously described [42]. Peptides were analyzed using the UltiMate 3000 RSLCnano system connected with an Orbitrap Exploris 480 equipped with a FAIMS interface (Thermo Fisher Scientific). Samples were first loaded onto trap column (PepMap 100 C18, 3 μm, 75 μm×20 mm) using flow rate 5 µL/min and then separated on analytical column (PepMap RSLC C18, 2 μm, 75 μm×250 mm; both columns from Thermo Fisher Scientific) by running a linear gradient (2% ACN/0.1% FA as phase A; 80% ACN/0.1% FA as phase B) from 2 to 34.5% B in 25 min and from 34.5 to 45% B in 5 min at a flow rate of 250 nL/min. MS analysis was done using data-dependent acquisition with FAIMS CV set to -50 V. MS spectra in the 350–1200 *m/z* range were acquired at a resolution of 120,000 with a normalized AGC target set to 300%. Multiply charged precursor ions with the minimal intensity of 5000 and not fragmented during the previous 17 s were isolated by 1 *m/z* window and taken for higher collisional dissociation (HCD) with normalized collision energy set to 28. MSMS spectra were acquired at 30,000 resolution with a normalized AGC target of 100% and a maximal injection time of 54 msec. The sample obtained from < 15 kDa band was further re-analyzed by a longer LC method (linear gradient from 2 to 34.5% B in 85 min and from 34.5 to 45% B in 10 min) and MS acquisition using oscillating FAIMS CVs of -45 V and − 65 V. Data were interpreted using MSFragger (v4.1) [43]. Methionine oxidation and N-terminal acetylation were set as variable, and carbamidomethylation of cysteine was set as a fixed modification. Data were searched against the Human Reference proteome database (UniProt) combined with sequences of APP_Swe/Ind_ variant and Aβ fragment. Proteins were filtered at 1% FDR.

### Size-exclusion chromatography

The molecular weight of the Aβ42 peptide derived from APP_Swe/Ind_ conditioned medium was determined by size-exclusion chromatography using an NGC Medium-Pressure Chromatography System (Bio-Rad, USA) equipped with a Superdex 200, 10/300 GL column (Cytiva, USA). Phosphate-buffered saline (PBS, pH 7.4) was used as the mobile phase, using the 0.7 mL/min flow rate and 0.35 mL injection volume. The samples were separated into 28 fractions, followed by immunoblotting detection using an anti-Aβ antibody. The fractions with positive antibody detection were evaluated based on a calibration curve generated using a Cytiva low molecular weight gel filtration calibration kit (28403841).

### Statistical analysis

Data were calculated as mean ± SD from single experiments. Statistical analyses were performed using GraphPad Prism 9 software (GraphPad Software, San Diego, CA). Ordinary one-way ANOVA with Tukey’s post hoc test was employed, and a *p-*value < 0.05 was considered statistically significant.

## Results

### Generation of stable cell models overexpressing HSD10 and APP_Swe/Ind_

Besides its physiological functions, mitochondrial enzyme HSD10 has been linked to several neurodegenerative diseases, including AD, Parkinson’s disease, and HSD10 disease. In AD, HSD10 has been reported to exacerbate the Aβ pathology, leading to enhanced cell stress and mitochondrial dysfunction [9, 24].

**Fig. 2:**
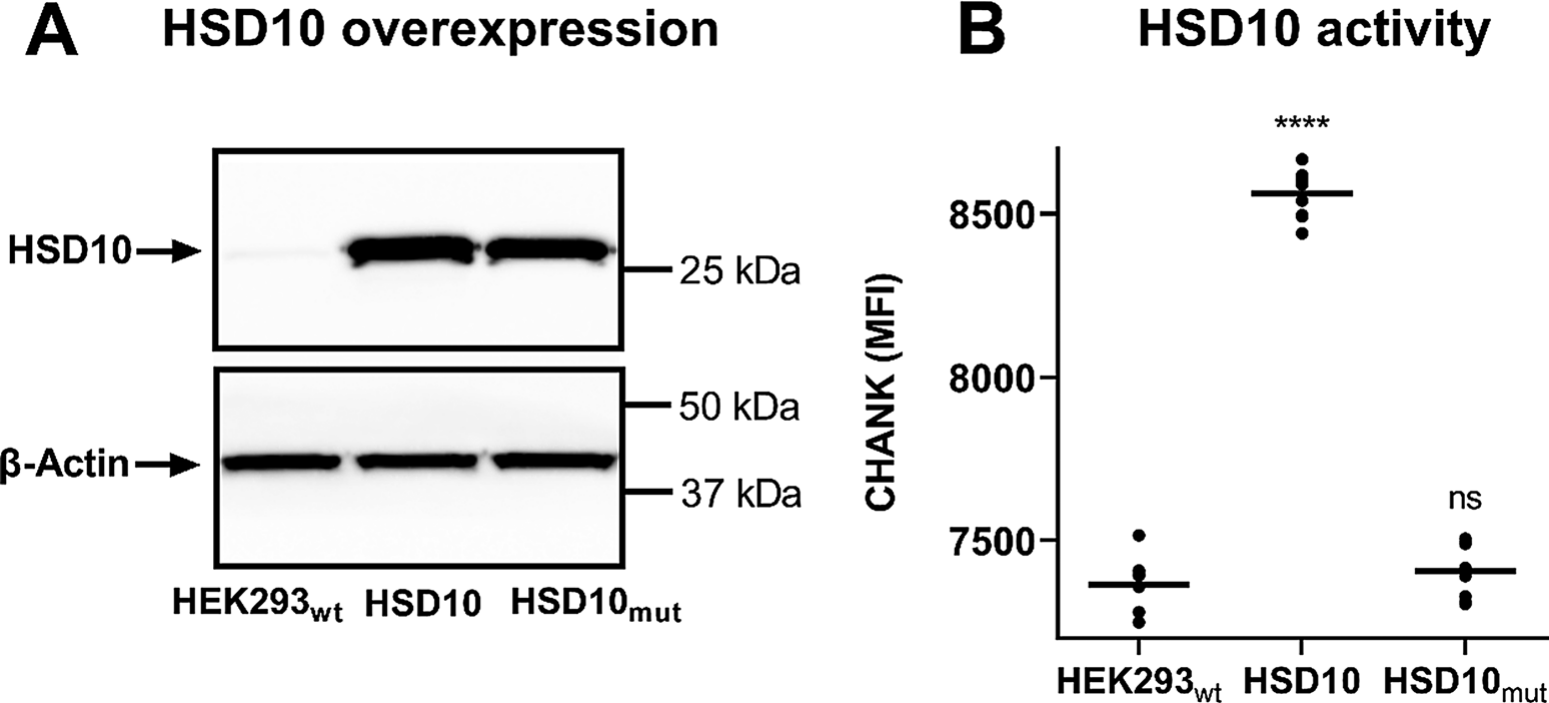
HSD10 overexpression in HSD10 and HSD10_mut_ cells. (**A**) Immunoblotting analysis in HEK293_wt_, HSD10, and HSD10_mut_ cells. (**B**) HSD10 activity determination via (-)-CHANA to CHANK turnover in HEK293_wt_, HSD10, and HSD10_mut_ cells (Ex/Em = 380/525nm). Statistical difference: *p ≤ 0.05, **p ≤ 0.01, ***p ≤ 0.001, ****p ≤ 0.0001 compared to the HEK293_wt_ control group

**Fig. 3:**
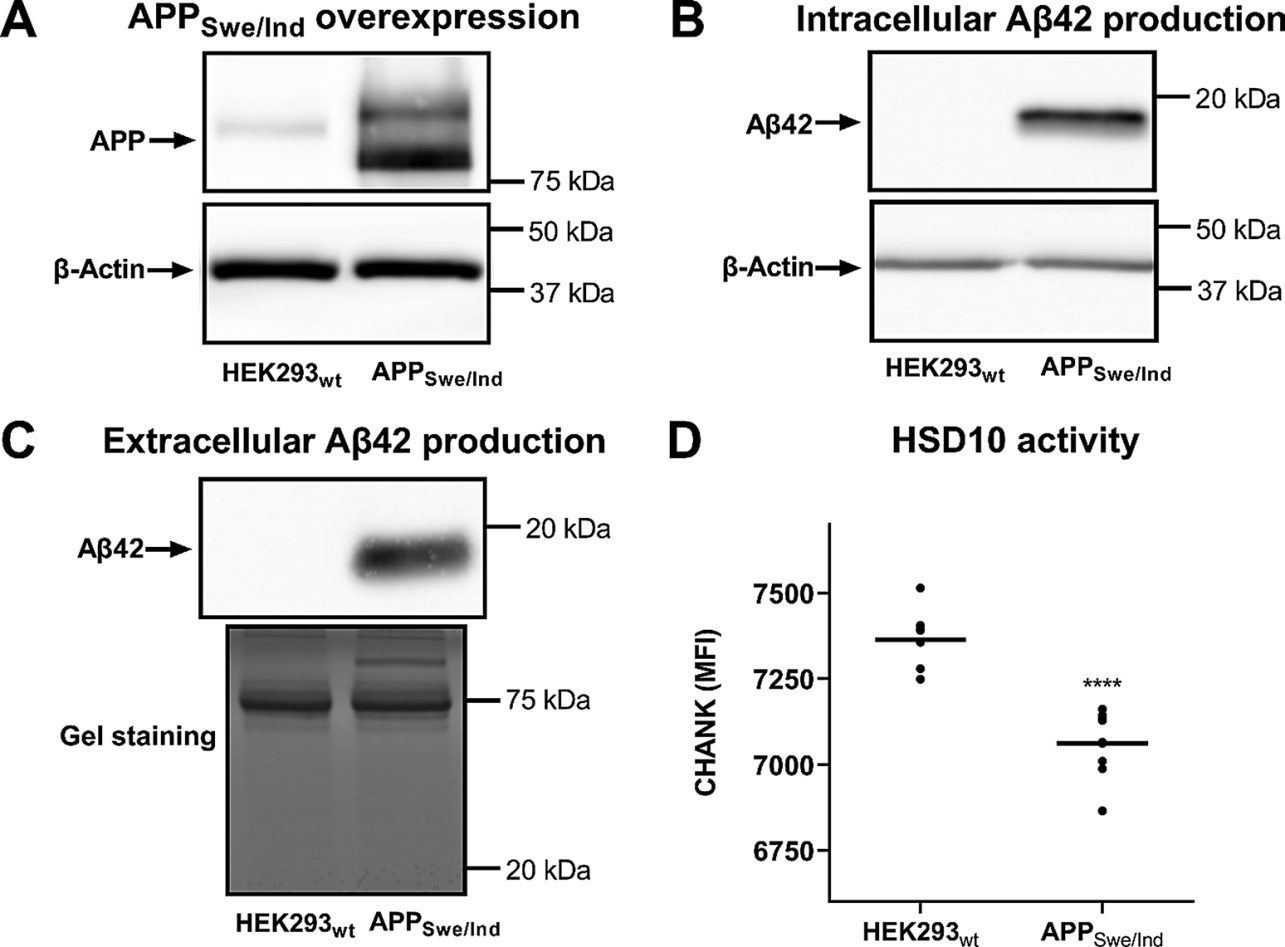
APP overexpression and Aβ production in APP_Swe/Ind_ cell line. (**A**) Immunoblotting analysis of APP expression in HEK293_wt_ and APP_Swe/Ind_ cells. (**B**) Immunoblotting analysis of Aβ42 production in HEK293_wt_ and APP_Swe/Ind_ cells. (**C**) Immunoblotting analysis of Aβ42 secretion to cultivation medium by HEK293_wt_, and APP_Swe/Ind_ cells (non-concentrated sample detection). (**D**) HSD10 activity determination via (-)-CHANA to CHANK turnover in HEK293_wt_, and APP_Swe/Ind_ cells (Ex/Em = 380/525nm). Statistical difference: *p ≤ 0.05, **p ≤ 0.01, ***p ≤ 0.001, ****p ≤ 0.0001 compared to the HEK293_wt_ control group

To investigate the role of the HSD10 in AD-related pathogenesis, stably-expressing monoclonal cell lines of human embryonic kidney HEK293 cells were created, overexpressing either HSD10 (referred to as HSD10 cells; Fig. 2A, Supplemental Figure S1-S2) or APP_Swe/Ind_ protein (amyloid-β precursor protein isoform 695 harbouring Swedish and Indiana mutations; referred to as APP_Swe/Ind_ cells; Fig. 3A, Supplemental Figures S4-S5). These cell lines allowed the description of the consequences of individual overexpression in detail.

In addition, a catalytically inactive HSD10 mutant cell line (Y168, K172– mutations in the catalytic active site [26, 44]; referred to as HSD10_mut_; Fig. 2A, Supplemental Figures S1-S2) was also constructed to evaluate the importance of the HSD10 enzymatic activity in related processes. The loss of enzyme activity was confirmed using the recombinant HSD10 enzyme and 17β-estradiol (E2) as a substrate (data not shown).

### The cellular catalytic activity of HSD10

In addition to its enzymatic function, the HSD10 protein is a structural component of the mitochondrial tRNA processing complexes [2, 4]. To differentiate between the enzymatic and non-enzymatic roles of the HSD10, the monoclonal HSD10 and HSD10_mut_ cells were assessed for their ability to convert the steroid-like (-)-CHANA fluorogenic probe [41], a specific substrate for HSD10 enzyme in the cellular environment.

The HSD10 cells exhibited elevated conversion of the probe, implying increased HSD10 enzyme activity compared to the HEK293_wt_ (Fig. 2B). In contrast, the HSD10_mut_ cells failed to convert the fluorogenic probe (Fig. 2B, Supplemental Figure S3) despite comparable expression levels of HSD10 protein (Fig. 2A, Supplemental Figures S1 and S2) in both HSD10 and HSD10_mut_ cell lines. These results confirm that mutations in the active site of HSD10 abolish its catalytic functions [10].

### Characterization of Aβ42 production by APP_Swe/Ind_ cells

Given the central role of Aβ in the pathogenesis of AD, Aβ production and release were investigated in the APP_Swe/Ind_ cells (Fig. 3A, Supplemental Figures S4-S5). The Aβ peptide was detected intracellularly (Fig. 3B, Supplemental Figure S6) and in the APP_Swe/Ind_ cell-conditioned medium (Fig. 3C, Supplemental Figures S7-S8), demonstrating that Aβ was both produced and extracellularly secreted (in the nanomolar range) by APP_Swe/Ind_ cells.

To verify the production of the specific Aβ42 fragment, which is expected as a result of the Swedish and Indiana mutations, two different antibodies for Aβ immunodetection were used. A signal around 20 kDa was observed for the antibody binding to all Aβ lengths (clone D54D2), whereas no signal was observed for the monoclonal antibody specific for Aβ40 (clone 32A1; data not shown), ruling out the possibility of Aβ40 production.

**Fig. 4:**
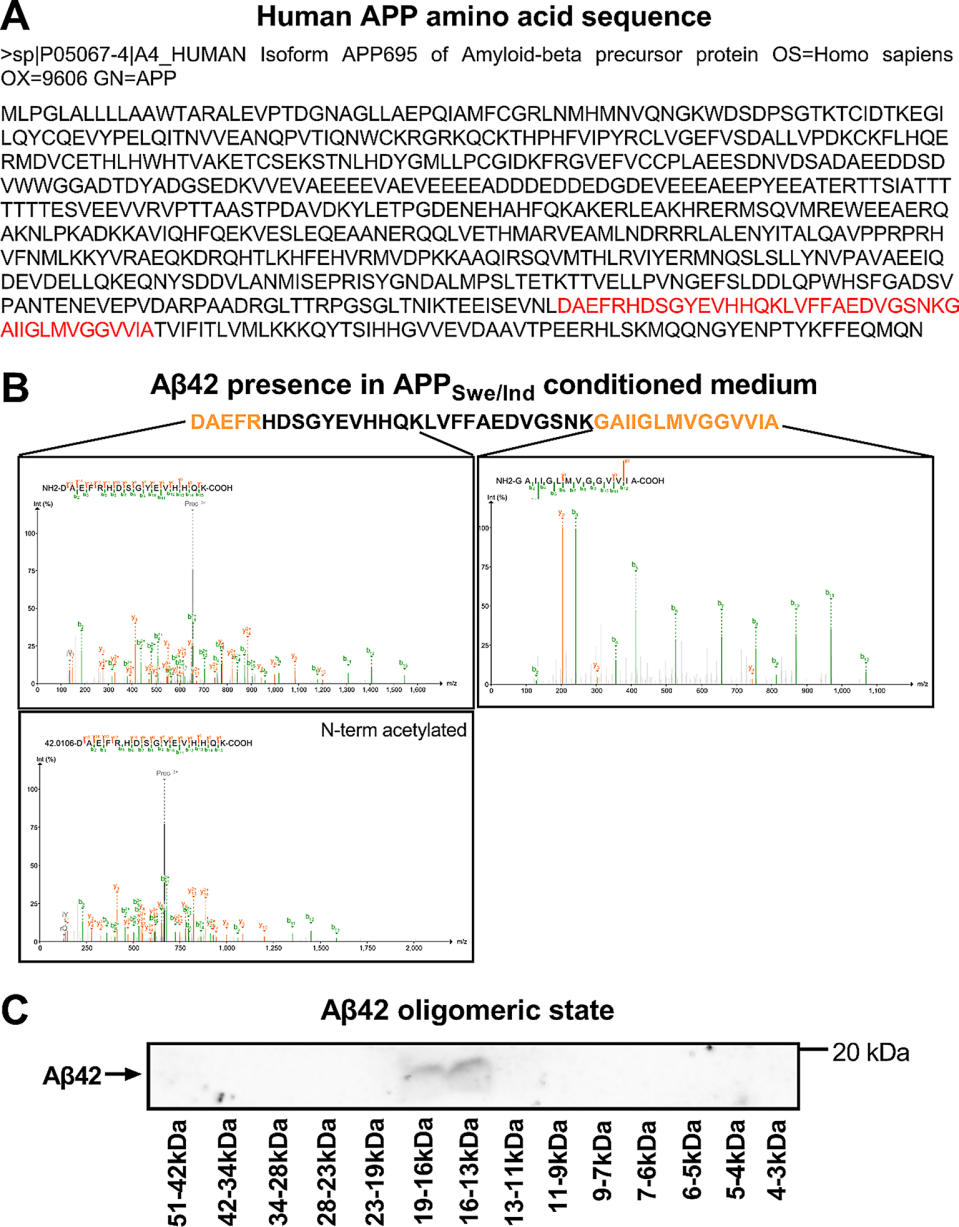
Cell-derived Aβ42 analysis. (**A**) The human APP_Swe/Ind_ amino acid sequence from the human reference proteome FASTA file (Uniprot) used in the proteomic data search. The sequence of Aβ42 is highlighted in red. (**B**) Confirmation of Aβ42 presence in APP_Swe/Ind_ conditioned medium. Proteins isolated from the medium were separated by SDS-PAGE and digested by trypsin. Tryptic peptides specific for Aβ42 (yellow), and not for APP precursor, were identified only in gel band corresponding to < 20 kDa (Band E of Supplemental Figure S9A). (**C**) Western blot analysis of separated fractions of APP_Swe/Ind_ cells’ conditioned medium retrieved from size-exclusion chromatography

Mass-spectrometry further confirmed the presence of Aβ42 by identifying Aβ42-specific peptides in the tryptic digest of gel-separated APP_Swe/Ind_ conditioned serum and protein-free media (Supplemental Figure S9A, Band E). Moreover, N-terminal acetylation of Aβ42 was identified (aspartic acid, D; Fig. 4A and B), indicating post-cleavage processing of Aβ42 peptide and further strengthening the evidence of intracellular Aβ42 production. In addition to Aβ42, full-length APP_Swe/Ind_ and APP-derived N-terminal cleavage fragments (sAPPβ) were detected in the conditioned medium, as indicated by the sequence coverage (Supplemental Figure S9B-C).

The oligomeric state of the cell-derived extracellular Aβ42 was determined using size-exclusion chromatography followed by the immunodetection of separated fractions. The most abundant detection of the Aβ42 was revealed under 20 kDa (between 13 and 19 kDa) (Fig. 4C, Supplemental Figures S10-S11), suggesting the formation into oligomers rather than remaining in monomeric form (4.5 kDa).

### Evaluation of the Aβ42 impact on endogenous HSD10 enzymatic activity

The cell-based production of Aβ42 prompted an investigation into whether intracellular Aβ42 affects the enzymatic function of endogenous HSD10. Experiments using a fluorogenic probe demonstrated that APP_Swe/Ind_ cells have reduced HSD10 enzymatic activity compared to the HEK293_wt_ cell line (Fig. 3D, Supplemental Figure S3). These results indicate that APP_Swe/Ind_ overexpression and the associated Aβ42 production are linked to decreased endogenous HSD10 enzymatic activity.

### Toxicity and mitochondrial impairment in HSD10 and APP_Swe/Ind_ cells

**Fig. 5:**
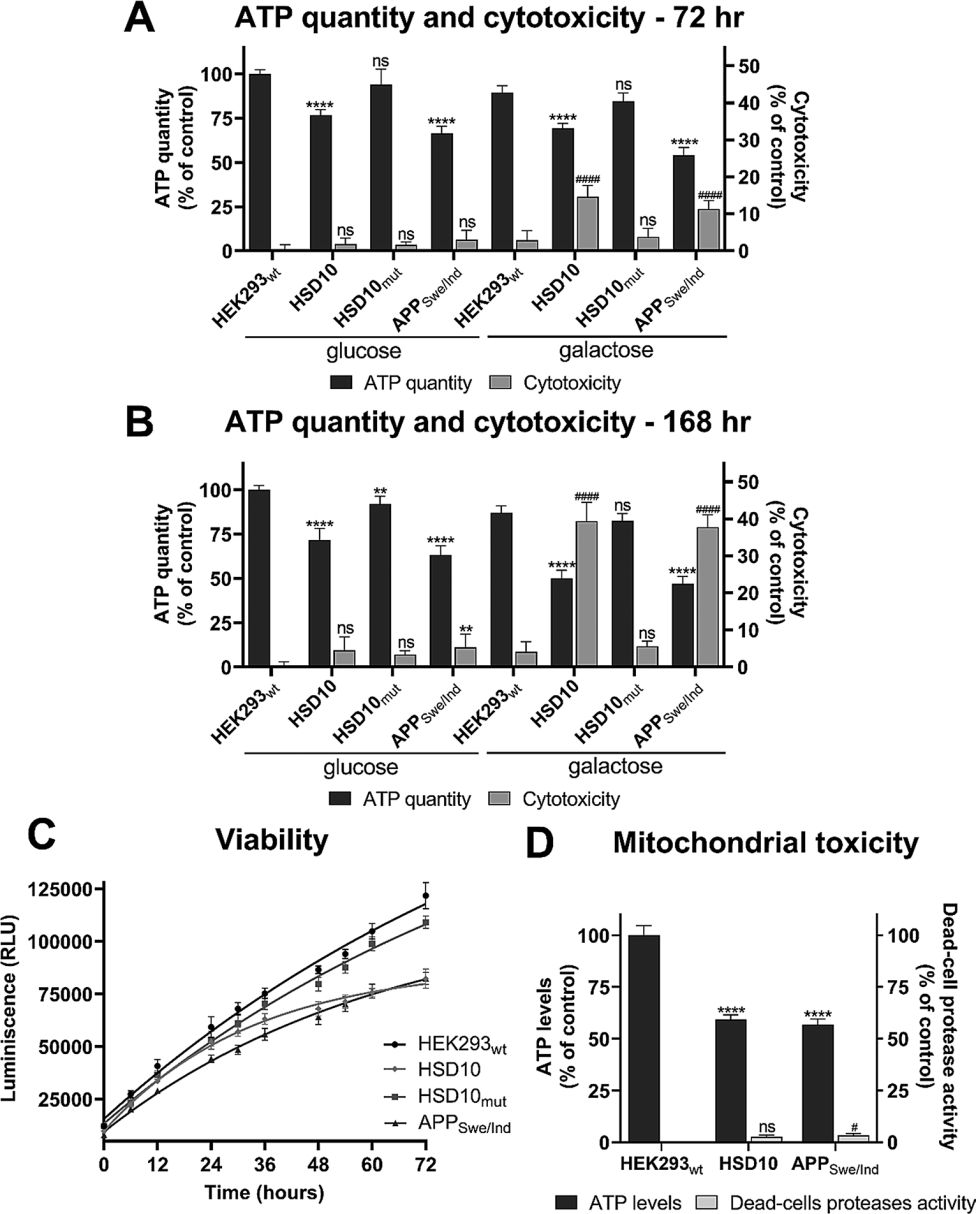
Cell and mitochondrial fitness parameters in HEK293_wt_, HSD10, HSD10_mut_, and APP_Swe/Ind_ cells. (**A**) ATP levels and cytotoxicity in HEK293_wt_, HSD10, HSD10_mut_, and APP_Swe/Ind_ cell lines 72 hr post-seeding into the glucose and galactose media. Data were normalized between DMSO-treated (1%) and valinomycin-treated (100 µM) HEK293_wt_ cells cultivated in a glucose medium. Values are given as means ± SD from three independent cell culture preparations with four technical replicates (*n* = 12). (**B**) ATP levels and cytotoxicity in HEK293_wt_, HSD10, HSD10_mut_, and APP_Swe/Ind_ cell lines 168 hr post-seeding into the glucose and galactose media. Data were normalized between DMSO-treated (1%) and valinomycin-treated (100 µM) HEK293_wt_ cells cultivated in a glucose medium. Values are given as means ± SD from three independent cell culture preparations with four technical replicates (*n* = 12). (**C**) Viability of HEK293_wt_, HSD10, HSD10_mut_, and APP_Swe/Ind_ cell lines monitored for 72 hr cultivation in galactose medium. Values are given as means ± SD from three independent cell culture preparations with three technical replicates (*n* = 9). (**D**) Mitochondrial toxicity in HEK293_wt_, HSD10, and APP_Swe/Ind_ cell lines 72 hr post-seeding into galactose medium. Data were normalized between DMSO-treated (1%) and valinomycin-treated (100 µM) (for dead-cells protease activity) or antimycin A-treated (1 µM) (for ATP levels measurements) HEK293_wt_ cells cultivated in galactose medium. Values are given as means ± SD from three independent cell culture preparations with four technical replicates (*n* = 12). SD: standard deviation. Statistical difference for ATP quantity and ATP levels: *p ≤ 0.05, **p ≤ 0.01, ***p ≤ 0.001, ****p ≤ 0.0001 compared to the HEK293_wt_ control group cultivated in glucose or galactose medium. Statistical difference for cytotoxicity and dead-cell protease activity: ^#^*p* ≤ 0.05, ^##^*p* ≤ 0.01, ^###^*p* ≤ 0.001, ^####^*p* ≤ 0.0001 compared to the HEK293_wt_ control group cultivated in glucose or galactose medium

In AD research, the effect of HSD10 overexpression on cell fitness has been primarily studied in an Aβ-rich environment [24, 26, 44]. However, HSD10 overexpression may also impact cell fitness through independent mechanisms, which could contribute to AD pathology beyond Aβ. This study evaluated the effect of individual HSD10, HSD10_mut_, and APP_Swe/Ind_ overexpression on cellular fitness by assessing ATP levels, cytotoxicity, and cell viability. ATP quantification performed in a complete glucose medium at 72 and 168 hr after cell seeding revealed significantly reduced ATP levels in HSD10 and APP_Swe/Ind_ cells, whereas HSD10_mut_ cells showed unaltered ATP levels. Cytotoxicity was found to be insignificant in all three cell lines when compared to control HEK293_wt_ cells, even at the late interval (Fig. 5A and B, Supplemental Figure S12), and all cell lines remained viable during long-term cultivation in complete glucose medium (data not shown).

Surprisingly, the absence of cytotoxicity in HSD10 and APP_Swe/Ind_ overexpressing cells contrasts with previous reports indicating that Aβ peptide affects mitochondrial processes and leads to cell viability decline [45, 46]. Since both HSD10 and APP_Swe/Ind_ overexpression are thought to affect mitochondrial function [24, 26, 46], we next assessed cell impairment by altering the major carbon source (galactose instead of glucose). This shift was reported to expose underlying mitochondrial damage not visible in high-glucose conditions [47–51]. Both HSD10 and APP_Swe/Ind_ cells cultured in galactose medium showed a further decrease in ATP levels compared to glucose medium, accompanied by the onset of cytotoxicity as early as 72 hr and worsening at 168 hr (Fig. 5A and B, Supplemental Figure S12). Real-time cell viability measurement confirmed a significant reduction in viable cells for both HSD10 and APP_Swe/Ind_ cells compared to HEK293_wt_ after 72 hr in galactose medium (Fig. 5C, Supplemental Figure S13). In contrast, HSD10_mut_ cells were unaffected by the carbon source change and showed similar growth and ATP levels as HEK293_wt_ cells (Fig. 5A and B, and 5C, Supplemental Figures S12-S13). Findings indicate that HSD10 enzymatic activity is primarily responsible for HSD10-induced cellular damage. Notably, HEK293_wt_ cells exhibited no signs of cytotoxicity in galactose medium, confirming that the observed pathology was primarily due to HSD10 and APP_Swe/Ind_ overexpression, ruling out galactose toxicity as a potential contributor.

Since the cytotoxic effects of HSD10 and APP_Swe/Ind_ overexpression were observed exclusively in the galactose environment, mitochondrial dysfunction in these cells was further investigated. A mitochondrial toxicity assay was performed by measuring ATP levels (as an indicator of mitochondrial damage) and the activity of dead-cell proteases (as an indicator of secondary toxicity, such as necrosis) in glucose-free conditions. After 72 hr of cultivation in a galactose medium, both HSD10 and APP_Swe/Ind_ cells showed a more than 40% decrease in ATP levels, accompanied by negligible dead-cell protease activity when compared to the HEK293_wt_ cells (Fig. 5D, Supplemental Figure S14). This suggests mitochondrial dysfunction, defined by a decrease of more than 20% in ATP levels and less than a 20% increase in dead-cell protease activity relative to control. These results confirm mitochondrial damage in both HSD10 and APP_Swe/Ind_ cell lines, leading to cytotoxicity and reduced cell viability in a glucose-free environment. Although these findings confirm mitochondrial-based toxicity in both models, the mechanisms underlying the observed toxicities require further investigation.

### Metabolic profiling of HSD10 and APP_Swe/Ind_ cells

**Fig. 6:**
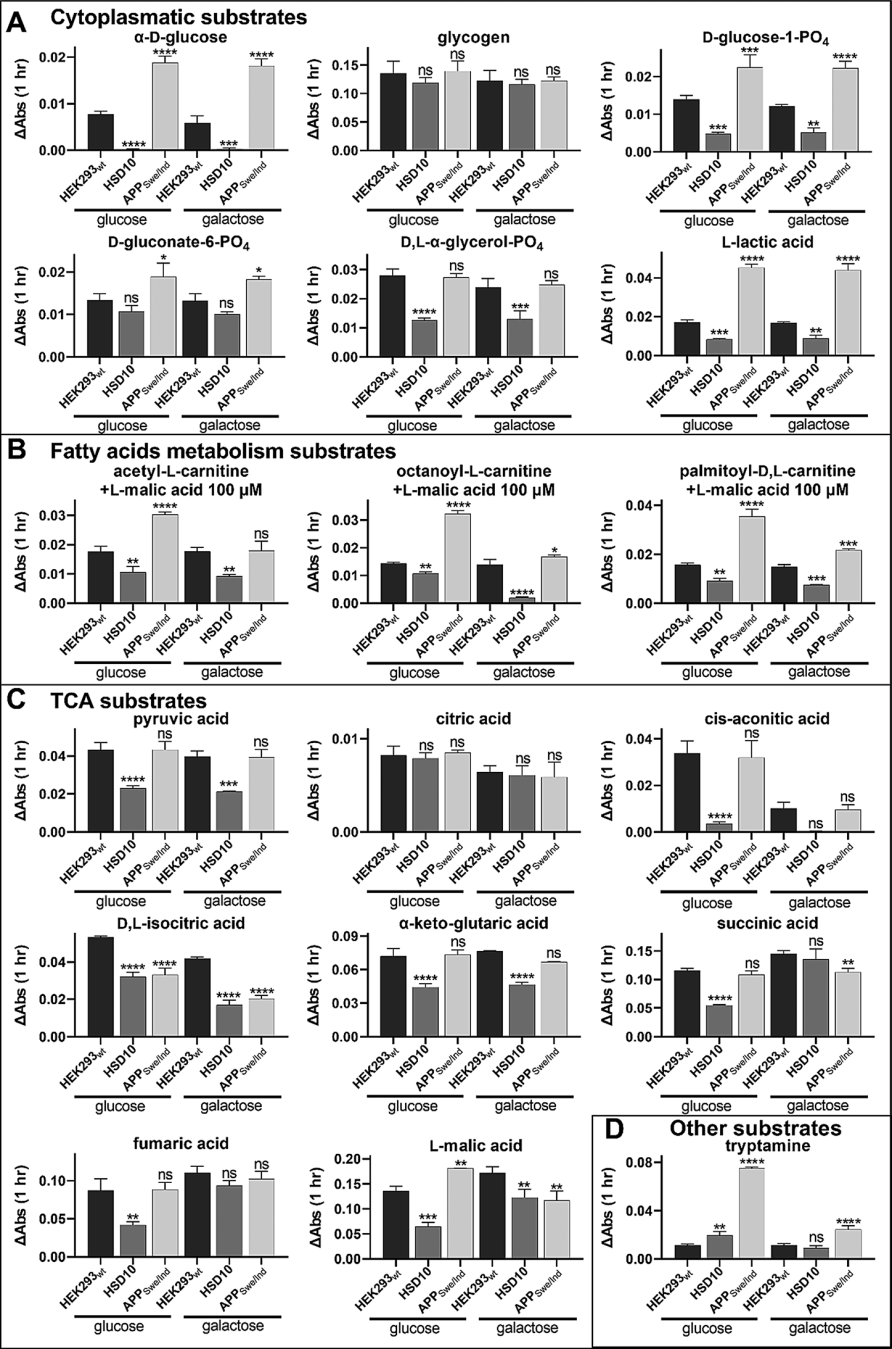
The mitochondrial electron flow changes (corresponding to metabolic changes) in HEK293_wt_, HSD10, and APP_Swe/Ind_ cells measured by absorbance (OD_590_) using MitoPlate S-1 assay. Substrate consumption rates were expressed as ΔAbs/hour from the linear phase. Conversion of cytoplasmatic substrates (**A**), fatty acid metabolism substrates (**B**), TCA cycle substrates (**C**), and other substrates (**D**) in HEK293_wt_, HSD10, and APP_Swe/Ind_ cells. Values are given as means ± SD from three independent cell culture preparations with one technical replicate (*n* = 3). SD: standard deviation. Statistical difference: *p ≤ 0.05, **p ≤ 0.01, ***p ≤ 0.001, ****p ≤ 0.0001 compared to the HEK293_wt_ control group

To gain deeper insight into the metabolic basis of the observed cell pathology, metabolic phenotypes of HSD10 and APP_Swe/Ind_ cells were analyzed using the MitoPlates S-1 assay, which evaluates mitochondrial electron flow (MEF) in the presence of various metabolic substrates. The results revealed markedly different patterns of MEF changes between the two cell lines (Fig. 6; Supplemental Figures S15 and S19), indicating distinct underlying mitochondrial alterations.

HSD10 cells exhibited a broad suppression of metabolism. Specifically, decreased MEF was observed in response to several glycolytic substrates, including α-D-glucose, D-glucose-1-PO_4_, D-gluconate-6-PO_4_, D, L-α-glycerol-PO_4_, and L-lactic acid (Fig. 6A; Supplemental Figure S15), suggesting impaired glycolytic input into mitochondrial respiration. In addition, a reduced MEF response to pyruvic acid and multiple TCA cycle intermediates (Fig. 6C) indicated a substantial disruption of the TCA cycle. Consistent with this, HSD10 cells also showed a marked decrease in basal oxygen consumption, which points to impaired mitochondrial respiration and supports the idea that TCA cycle dysfunction significantly contributes to reduced mitochondrial function (Methodology and Results in the Supplementary Information, Supplemental Figure S20). Furthermore, β-oxidation also appeared diminished, with lower MEF levels in response to various fatty acid substrates compared to HEK293_wt_ cells (Fig. 6B).

In contrast, APP_Swe/Ind_ cells displayed an overall increase in MEF in response to several glycolytic substrates, such as α-D-glucose, D-glucose-1-PO_4_, D-gluconate-6-PO_4_, and L-lactic acid (Fig. 6A), indicating an apparent enhancement of glycolytic metabolism. For β-oxidation substrates, these cells showed increased MEF levels, suggesting upregulation of fatty acid metabolism (Fig. 6B). Regarding the TCA cycle, APP_Swe/Ind_ cells exhibited variable, substrate-specific MEF changes (Fig. 6C), indicating a more selective and less globally impaired influence on TCA cycle activity compared to HSD10 cells.

Interestingly, both cell lines demonstrated increased MEF in response to tryptamine (Fig. 6D), particularly after cultivation in glucose-containing media, suggesting enhanced monoamine oxidase (MAO) activity. This effect was more pronounced in APP_Swe/Ind_ cells. Since elevated MAO activity is associated with increased ROS production [52], a phenomenon observed in AD [53, 54], it may contribute to the downstream oxidative stress and neuroinflammatory responses implicated in disease progression.

### Impact of cell-derived Aβ42 on HSD10 enzymatic activity and cytotoxicity in HSD10 cells

Most studies on Aβ have used synthetic peptides [44, 55], but these do not fully capture the complexity of naturally occurring Aβ. In this study, we used cell-derived Aβ42 from the APP_Swe/Ind_ cells to better reflect the physiological conditions found in AD. We aimed to explore how cell-derived Aβ42 affects HSD10 activity and contributes to HSD10-driven cytotoxicity.

**Fig. 7:**
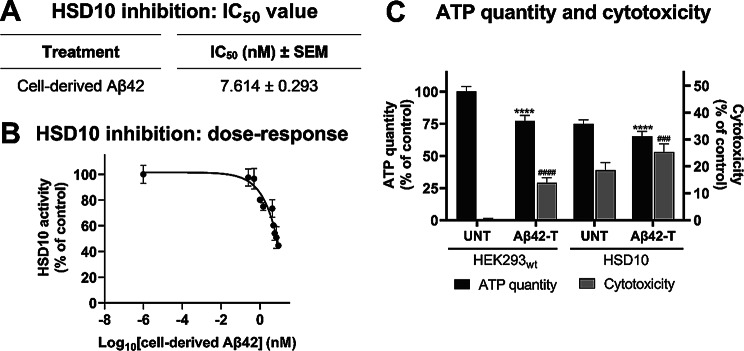
The effects of cell-derived Aβ42 treatment. (**A**) HSD10 inhibition in the cellular environment. The IC_50_ determination of cell-derived Aβ42 using (-)-CHANA fluorogenic probe in HSD10 cells in glucose medium. Values are given as means ± SEM from three cell culture preparations with three technical replicates (*n* = 9). (**B**) Dose-response curve showing inhibition of HSD10 activity in HSD10 cells by cell-derived Aβ42, measured using the fluorogenic probe (-)-CHANA. Cells were treated with Aβ42 at concentrations ranging from 0 to 9 nM in a glucose medium. Values are given as means ± SEM from three cell culture preparations with three technical replicates (*n* = 9). (**C**) ATP levels and cytotoxicity in HEK293_wt_ and HSD10 cells performed 72 hr post-seeding into galactose medium containing 7.6 nM cell-derived Aβ42. Data were normalized between DMSO-treated (1%) and valinomycin-treated (100 µM) HEK293_wt_ cells cultivated in galactose medium. Values are given as means ± SD from three independent cell culture preparations with three technical replicates (*n* = 9). SD: standard deviation. Statistical difference for ATP quantity: *p ≤ 0.05, **p ≤ 0.01, ***p ≤ 0.001, ****p ≤ 0.0001 compared to the untreated HEK293_wt_ or HSD10 control group. Statistical difference for cytotoxicity: ^#^*p* ≤ 0.05, ^##^*p* ≤ 0.01, ^###^*p* ≤ 0.001, ^####^*p* ≤ 0.0001 compared to the untreated HEK293_wt_ or HSD10 control group

To investigate the impact of cell-derived Aβ42 on HSD10 activity, APP_Swe/Ind_ conditioned glucose medium (as a source of Aβ42) was prepared and used for the treatment of HSD10 cells. The treatment in glucose conditions significantly reduced HSD10 activity (Fig. 7B), as measured by a fluorogenic probe, with an IC_50_ value of 7.6 nM (Fig. 7A). These findings demonstrate that cell-derived Aβ42 effectively reduces HSD10 activity and can be used as an effective Aβ preparation.

To assess the effects of cell-derived Aβ42 on cell fitness, a fresh conditioned galactose medium containing Aβ42 was prepared and used for the treatment of HSD10 and HEK293_wt_ cells. After 72 hr of exposure to 7.6 nM Aβ42 in this galactose medium, both cell lines exhibited decreased ATP levels and increased cytotoxicity (Fig. 7C, Supplemental Figure S16). Specifically, in HEK293_wt_ cells, there was a 23% decrease in ATP levels and a 14% increase in cytotoxicity noted. The HSD10 cells exposed to cell-derived Aβ42 exhibited a further 10% reduction in ATP levels, with an additional 7% increase in cytotoxicity compared with untreated HSD10 cells.

These results suggest that cell-derived Aβ42 impacts HSD10 enzymatic activity and exacerbates the cytotoxic effects associated with HSD10 overexpression, providing further evidence of the role of Aβ in modulating HSD10-driven toxicity.

### HSD10 inhibitors’ impact on cell viability and HSD10 activity

**Fig. 8:**
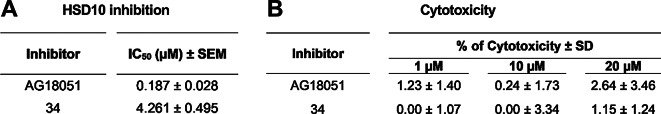
Inhibitors **AG18051** and **34** characterizations. (**A**) HSD10 inhibition in the cellular environment. The IC_50_ determination of inhibitors **AG18051** and **34** using (-)-CHANA fluorogenic probe in HSD10 cells in glucose medium. Values are given as means ± SEM from three cell culture preparations with three technical replicates (*n* = 9). (**B**) Cytotoxicity in HEK293_wt_ after 48 hr of treatment with **AG18051** or inhibitor **34** (1–20 µM) in galactose medium. Data were normalized between DMSO-treated (1%) and valinomycin-treated (100 µM) HEK293_wt_ cells cultivated in galactose medium. Values are given as means ± SD from two independent cell culture preparations with four technical replicates (*n* = 12). SEM: standard error of the mean. SD: standard deviation

The concept of HSD10 inhibition as a potential treatment for HSD10 overexpression associated with Alzheimer’s disease pathology was introduced many years ago [10, 24, 38, 44]. To study the effects of cellular HSD10 inhibition in the context of reducing HSD10-driven pathology, two nanomolar HSD10 inhibitors with different mechanisms of inhibition were employed: the irreversible inhibitor **AG18051** [38] and the reversible benzothiazole inhibitor **34** [39] (both structures shown in Fig. 1A). Both inhibitors were previously shown to inhibit the recombinant HSD10 enzyme [31]. Here, we confirmed their activity in a cellular environment using the fluorogenic probe (-)-CHANA, determining IC_50_ values of 0.19 µM for **AG18051** and 4.26 µM for inhibitor **34** (Fig. 8A).

Since both inhibitors were previously shown to have no cytotoxic effects in a glucose environment [31], they were retested in a glucose-free environment to exclude the possibility of hidden cytotoxicity that may have affected the results. Cytotoxicity assessment, 48 hr post-treatment, in HEK293_wt_ cells cultured in galactose medium, revealed no signs of cytotoxicity for either inhibitor, even at concentrations up to 20 µM (Fig. 8B), indicating their safe use in cell treatments.

### Evaluation of HSD10 inhibitors in reducing HSD10-driven pathology

Recent HSD10 inhibitors have primarily been tested on recombinant HSD10 enzymes to assess their binding and inhibitory properties. To extend the understanding of HSD10 inhibition, this study evaluated the potential of these inhibitors to mitigate the pathological effects of HSD10 overexpression. HSD10 cells were cultured in a galactose medium and treated with various concentrations of the inhibitors (ranging from 0.5 to 4 times their IC_50_ values) for 72 hr.

**Fig. 9:**
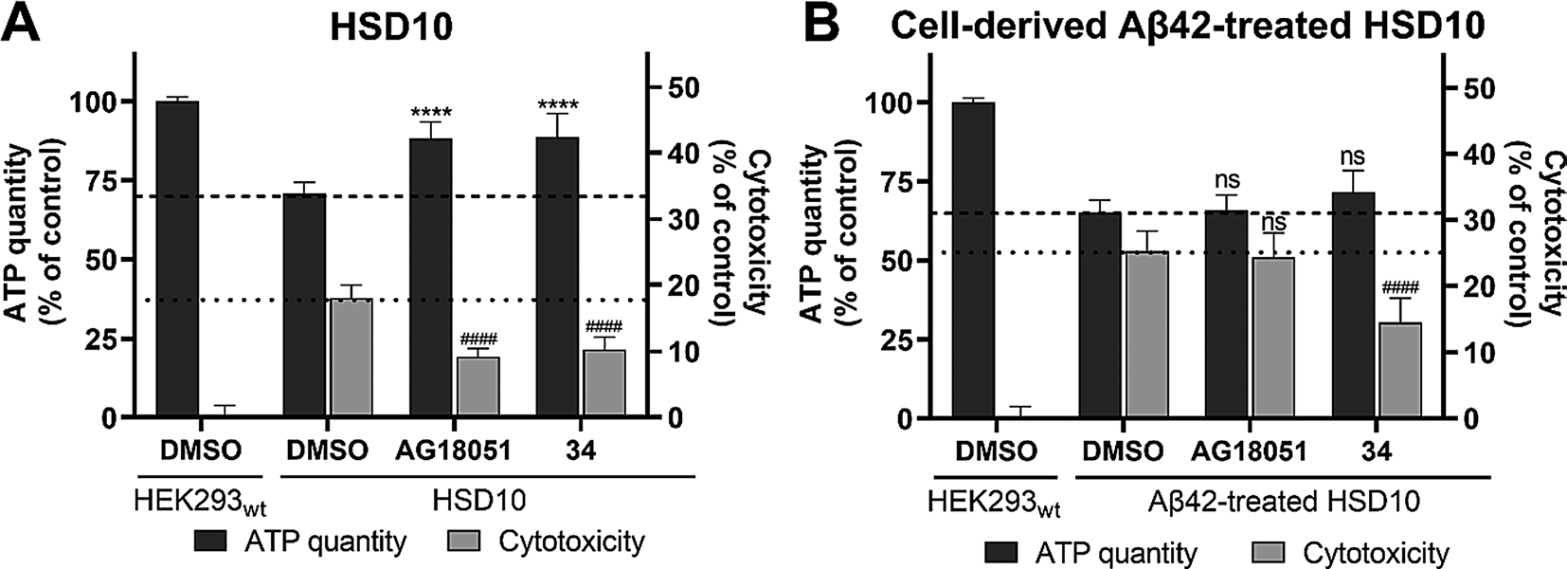
The ability of inhibitors **AG18051** and **34** to reduce HSD10-associated cytotoxicity. (**A**) ATP levels and cytotoxicity in HSD10 cells treated with HSD10 inhibitors (three times the IC_50_ value; 0.57 µM for **AG18051** or 12.78 µM for inhibitor **34**) were performed 72 hr post-seeding into galactose medium. Data were normalized between DMSO-treated (1%) and valinomycin-treated (100 µM) HEK293_wt_ cells cultivated in galactose medium. Values are given as means ± SD from three independent cell culture preparations with four technical replicates (*n* = 12). (**B**) ATP levels and cytotoxicity in HSD10 cells were measured 72 hr post-seeding into the Aβ42-containing galactose medium with HSD10 inhibitor co-treatment (three times the IC_50_ value; 0.57 µM for **AG18051** or 12.78 µM for inhibitor **34**). Data were normalized between DMSO-treated (1%) and valinomycin-treated (100 µM) HEK293_wt_ cells cultivated in galactose medium. Values are given as means ± SD from three independent cell culture preparations with three technical replicates (*n* = 9). SD: standard deviation. Statistical difference for ATP quantity: *p ≤ 0.05, **p ≤ 0.01, ***p ≤ 0.001, ****p ≤ 0.0001 compared to the untreated HSD10 control group. Statistical difference for cytotoxicity: ^#^*p* ≤ 0.05, ^##^*p* ≤ 0.01, ^###^*p* ≤ 0.001, ^####^*p* ≤ 0.0001 compared to the untreated HSD10 control group

Both inhibitors, at all tested concentrations, increased ATP levels and significantly reduced cytotoxicity in the HSD10 cells (Supplemental Figure S17). The most effective concentrations were three times the IC_50_ values (0.57 µM for **AG18051** and 12.78 µM for inhibitor **34**) (Fig. 9A, Supplemental Figure S17), which led to an 18% increase in ATP levels and nearly a halving decrease in cytotoxicity. These findings confirm the ability of HSD10 inhibitors to reduce the pathological effects associated with HSD10 overexpression.

### Evaluation of HSD10 inhibitors in an Aβ42-rich environment

To further explore the therapeutic potential of HSD10 inhibitors under disease-relevant conditions, their effects were tested in an Aβ42-enriched environment, which is known to exacerbate mitochondrial dysfunction and contribute to early neuronal damage in AD [56]. Given that Aβ42 exacerbates the decline in HSD10 cell fitness (Fig. 7B), we assessed whether treatment with **AG18051** or inhibitor **34** (at 3× IC_50_) could mitigate or reverse the pathological phenotype in HSD10 cells cultured in galactose medium supplemented with 7.6 nM cell-derived Aβ42, a concentration previously shown to impact HSD10 activity (Fig. 7A).

The results showed (Fig. 9B, Supplemental Figure S18) that treatment with inhibitor **AG18051** had no significant positive effect on improving pathological parameters in the Aβ42-rich environment, as both cytotoxicity and ATP production decline remained unchanged upon co-administration of **AG18051**. In contrast, treatment with inhibitor **34** led to a slight, but not statistically significant increase in ATP levels, from 65 to 72%, and a significant reduction in cytotoxicity, from 25 to 14%. Such a phenomenon was also confirmed by another viability assay (using resazurin) (Methodology and Results in the Supplementary Information, Supplemental Figure S21). These findings suggest that inhibitor **34** has the potential to partially reverse HSD10-driven pathology in an Aβ42-rich environment and highlight it as a promising candidate for therapeutic development in Alzheimer’s disease.

## Discussion

This study investigated the individual and combined effects of HSD10 overexpression and Aβ42 production on mitochondrial and cellular dysfunctions associated with AD. Using stably expressing monoclonal HEK293 cell lines, we provide new insights into how these two pathological factors contribute to cellular impairments relevant to the disease.

Our study demonstrates for the first time that HSD10 overexpression alone can induce cellular damage, evidenced by impaired cell viability, ATP production, and increased cytotoxicity (Fig. 5). Importantly, this effect depended on HSD10 enzymatic activity, as the catalytically inactive HSD10_mut_ variant did not cause similar impairments. This observation expands previous findings, which primarily linked HSD10 toxicity to its interaction with Aβ [10, 24, 26] and failed to show the cytotoxic effect of HSD10 overexpression alone in transgenic models [24, 44]. Our data suggest that HSD10 overexpression may independently drive neurodegenerative processes, making it a potential pathological contributor in AD and related disorders. These findings also highlight the importance of tightly regulated HSD10 expression levels, as increases may disrupt cellular homeostasis and lead to detrimental consequences.

Interestingly, HSD10-induced toxicity was only apparent under glucose-free conditions, which forces the cells to engage mitochondria. This observation aligns with previous reports showing that a glucose-rich environment may mask mitochondrial dysfunctions by using cellular aerobic glycolytic activity. When glucose is replaced by galactose, cells can no longer rely on glycolysis for ATP production and become entirely dependent on mitochondrial function for survival, revealing the hidden mitochondrial impairment [47–51]. This metabolic dependency may explain why previous models failed to detect HSD10-driven cytotoxicity and underscores the critical importance of metabolic context in uncovering mitochondrial pathologies.

Similarly, APP_Swe/Ind_ cells, which produce Aβ42, also exhibited cytotoxicity, reduced viability, and decreased ATP production (Fig. 5). Cytotoxic effects associated with APP_Swe/Ind_ and/or Aβ42 have been reported previously [45, 46]. Other APP cleavage products, particularly the C-terminal 99 amino acid long fragment (C99), have also been implicated in mitochondrial dysfunction and cellular toxicity [57]. However, in our study, the C99 fragment was not detected in the APP_Swe/Ind_ sample (Supplemental Figure S9), indicating that cytotoxicity observed in this model is unlikely to be mediated by C99 but more probably results from Aβ42 accumulation.

Notably, Aβ42-induced cytotoxicity in our model also became apparent only under glucose-free conditions (Fig. 5A and B). Importantly, this not only suggests mitochondrial damage due to Aβ42 production, but this phenomenon observed in both lines (APP_Swe/Ind_ and HSD10 cell lines) may be highly significant in the context of AD. Aging is associated with impaired glucose utilization [58], a hallmark also evident in AD brains [59]. Therefore, our findings raise the possibility that reduced glucose utilization may act as a triggering factor for developing neuronal defects observed in AD.

The observation that cytotoxicity in both overexpression models (APP_Swe/Ind_ and HSD10 cells) occurred exclusively under glucose-free conditions suggests that mitochondrial damage is a central mediator of the observed cytotoxicity. This mitochondrial dysfunction was confirmed in both cell lines (Fig. 5D). However, metabolic profiling using substrate utilization assay (MEF measurements) revealed distinct underlying mechanisms contributing to mitochondrial impairment in each cell line (Fig. 6).

In HSD10 cells, mitochondrial dysfunction was primarily characterized by disturbances in β-oxidation and TCA cycle activity. These alterations are likely a consequence of increased HSD10 enzymatic activity. HSD10 plays a crucial role in steroid metabolism, particularly by catalyzing the oxidation of E2 to estrone [27]. E2 has been shown to modulate β-oxidation: its supplementation increases β-oxidation capacity [60], induces expression of β-oxidation-related genes [61], and rescues β-oxidation gene expression deficits in knockout mouse models [62]. Therefore, enhanced HSD10 activity in our model likely increases E2 turnover, leading to reduced intracellular E2 levels. This E2 depletion may contribute to the diminished β-oxidation observed in HSD10 cells. A reduction in β-oxidation also decreases acetyl-CoA availability, thereby limiting substrate input into the TCA cycle [63], potentially explaining the TCA cycle downregulation seen in these cells (Fig. 6C).

Moreover, increased MEF during tryptamine utilization may reflect elevated MAO activity, which in turn could be indicative of enhanced oxidative stress. It is important to note that the MitoPlate assay predominantly measures mitochondrial activity by detecting NADH and FADH₂ production, which mainly reflects TCA cycle function. Thus, the pronounced alterations in the TCA cycle in HSD10 cells may interfere with accurate measurements of glycolytic activity. In this context, it remains uncertain whether the observed MEF patterns truly reflect decreased glycolytic metabolism or are instead artifacts of diminished TCA cycle activity.

In contrast, APP_Swe/Ind_ cells exhibited mitochondrial dysfunction driven by glucose hypermetabolism and partial TCA cycle impairment. These findings are consistent with previous studies suggesting that while late stages of AD are typically associated with glucose hypometabolism, hypermetabolism may occur in earlier stages [49, 64–68]. It has been proposed that cells respond to early Aβ-induced mitochondrial dysfunction by shifting from OXPHOS (oxidative phosphorylation) to aerobic glycolysis to maintain ATP production [49]. This metabolic adaptation may initially increase glucose utilization but ultimately becomes unsustainable, collapsing towards reduced glucose metabolism in the late stages of disease progression [49, 69].

Our data support previous reports suggesting that glucose metabolism may be upregulated in response to Aβ42 toxicity. In APP_Swe/Ind_ cells, this metabolic shift likely acts as a compensatory mechanism to mitigate mitochondrial dysfunction associated with partial TCA cycle impairment, thereby helping to maintain cellular energy homeostasis. Interestingly, β-oxidation also appeared upregulated in these cells, a response that may be linked to the same adaptive process, since a shift from OXPHOS to glycolysis has been shown to promote β-oxidation [70, 71]. Finally, similar to HSD10 cells, increased MEF during tryptamine utilization in APP_Swe/Ind_ cells suggests the presence of elevated oxidative stress.

While further studies will be necessary to assess the activity of individual metabolic enzymes directly, the significant differences in metabolic responses between the HSD10 and APP_Swe/Ind_ overexpression models strongly suggest that the resulting mitochondrial dysfunction arises through distinct mechanisms.

The secretion of Aβ42 into the conditioned medium of APP_Swe/Ind_ cells provided a physiologically relevant model for testing the effects of Aβ42. The use of cell-derived Aβ42 proved to be an effective preparation with a high capacity to inhibit both the endogenous HSD10 enzyme in APP_Swe/Ind_ cells (Fig. 3D) and the overexpressed HSD10 enzyme in HSD10 cells (Fig. 7A). Moreover, the cell-derived Aβ42 appeared to be more effective in inhibiting the overexpressed HSD10 than the synthetic Aβ42 preparation used in prior studies [41, 44]. This discrepancy is likely due to differences in oligomerization states. Synthetic preparations are prone to structural variability, complicating their characterization, and were reported to be less potent than cell-derived Aβ42, which appears to better mimic the biologically active species relevant to AD pathology [72]. Our results suggest that cell-derived Aβ42 used in this study predominantly forms trimers or tetramers, with molecular weights around 13–19 kDa (Fig. 4C), consistent with previously reported highly toxic Aβ species [73]. Although we cannot exclude contributions from other APP fragments present in the conditioned medium, only Aβ fragments have been shown to reduce recombinant HSD10 activity to date [10, 74–77]. Nonetheless, if other APP fragments also contribute to AD pathology, using cell-derived Aβ42 provides a more physiologically relevant context, reflecting the complex environment in the AD brain where multiple APP cleavage products coexist [78–81].

To explore the functional consequences of Aβ42-driven HSD10 inhibition, we examined cellular fitness parameters in HEK293_wt_ and HSD10 cells treated with cell-derived Aβ42 (Fig. 7C). The results revealed that Aβ42 negatively impacted cell fitness in both cell lines. In HEK293_wt_ cells, the observed decrease in ATP levels and the increase in cytotoxicity were very similar to the phenotype observed in APP_Swe/Ind_ cells (Fig. 5A), although a direct comparison is limited by the fact that Aβ42 concentration was not quantified in the APP_Swe/Ind_ cells. Measuring intracellular Aβ42 concentration is challenging due to rapid fluctuations in secretion levels, which can complicate accurate measurement [82]. Notably, HSD10-overexpressing cells exhibited an additional decrease in ATP levels and increased cytotoxicity compared to untreated HSD10 cells, suggesting that HSD10-driven impairment is exacerbated in an Aβ42-rich environment. Whether this exacerbation is a simple additive effect, synergistic toxicity resulting from direct interaction between HSD10 and Aβ42, or if additional mechanisms are involved, remains to be determined. Further studies are required to unravel the molecular basis of this interaction and its potential implications for mitochondrial dysfunction in AD.

The concept of HSD10 inhibition as a potential treatment for HSD10 overexpression associated with AD pathology was introduced several years ago [10, 24, 38, 44], and multiple classes of nanomolar inhibitors have since been developed [27, 30, 32]. To explore their therapeutic potential in a cellular context, we investigated two HSD10 inhibitors with distinct mechanisms of action: **AG18051** [38], an irreversible inhibitor that forms a covalent adduct with the NAD⁺ cofactor in the enzyme’s active site, and inhibitor **34** [39], a reversible benzothiazole-based compound that preferentially binds to the enzyme-substrate complex. Both inhibitors were previously shown to potently inhibit recombinant HSD10 with IC₅₀ values of 89 nM for **AG18051 **and 346 nM for inhibitor **34** [31] and to penetrate cells without inducing cytotoxicity [31]. Here, we determined their IC₅₀ values in the cellular environment to be 0.19 µM for **AG18051** and 4.26 µM for inhibitor **34** (Fig. 8A), further supporting their potential translational relevance. While most prior studies focused primarily on **AG18051** [38], this study represents the first comparative cellular characterization of both inhibitors and their potential to reduce HSD10-driven pathology, offering new insights into the therapeutic application of HSD10 inhibition in AD treatment.

The results of our study demonstrated that HSD10 inhibition can mitigate the pathological consequences of its overexpression (Fig. 9A). Despite their differing modes of action, both **AG18051** and inhibitor **34 **significantly improved cellular fitness in HSD10-overexpressing cells, supporting that the observed effects are directly attributable to HSD10 inhibition rather than off-target effects. While **AG18051** was previously evaluated in a similar cellular model of HSD10 overexpression [44], the study did not detect cytotoxic effects linked to HSD10 overexpression. However, it did report impaired mitochondrial respiration, which was restored after** AG18051** treatment. In contrast, our study confirms that HSD10 overexpression leads to measurable cytotoxicity and demonstrates that both inhibitors **AG18051** and **34 **can reverse this pathology. This also represents the first report on the use of inhibitor **34** in this context.

To extend the therapeutic relevance, we further evaluated the efficacy of both inhibitors in an Aβ42-rich environment (Fig. 9B), as Aβ42 is widely recognized as a key driver of mitochondrial dysfunction and early neuronal loss in AD [56]. While both inhibitors were effective in cells with HSD10 overexpression alone, only inhibitor **34** was able to rescue cellular fitness in the presence of Aβ42. In contrast, **AG18051** failed to reverse the exacerbated phenotype, suggesting its therapeutic potential may be limited in amyloidogenic conditions.

The different effects of **AG18051** and inhibitor **34 **raise essential questions about their mechanisms of action. Inhibitor **34** shares structural features with Thioflavin-T (a fluorescent Aβ-binding dye) and frentizole– compounds known to disrupt the interaction between Aβ and HSD10 [33]. Therefore, it is possible that **34 **not only inhibits HSD10 but also interferes with HSD10-Aβ binding or acts through direct binding to Aβ42. This additional effect may explain its greater potential in the Aβ42-rich environment. More research is needed to understand these mechanisms in detail. However, our results highlight the importance of testing inhibitors in disease-relevant conditions, not just for their binding and inhibition ability on isolated enzymes.

Overall, our study introduces a new approach to evaluating HSD10-targeted therapies in AD models. Importantly, we provide clear evidence that HSD10 overexpression is directly cytotoxic, supporting its role as a pathogenic factor in AD-related mitochondrial dysfunction. This finding represents a significant contribution to the field, as previous studies had not conclusively demonstrated this toxicity. Furthermore, we identify inhibitor **34** as a promising candidate, capable of reversing HSD10-driven pathology even in the presence of Aβ42. These findings support the idea that targeting HSD10 may help act against mitochondrial dysfunction in AD and reinforce the therapeutic potential of HSD10 inhibitors.

## Limitations

This study offers novel and valuable insights into HSD10 pathobiology within an Aβ42-rich environment. However, several limitations should be noted. First, we present a cellular model to clarify the pathological mechanisms associated with an Aβ42-rich environment; however, these experiments were conducted in HEK293 cells, which originate from embryonic kidney tissue and may not fully replicate neuronal metabolism. Nonetheless, these cells are widely used in Alzheimer’s disease research [83–86] and exhibit numerous neuronal characteristics, including the expression of over 60 neuron-associated genes such as neurofilament proteins, neuroreceptors, and ion channel subunits [87, 88]. Therefore, to strengthen the data, confirmation would require using another cell line or patient-derived cells. The second limitation pertains to the unknown mechanisms of action of inhibitor **34**, which could reveal more specific interactions involving this compound. In summary, further research is needed to clarify the specific mechanisms of both the inhibitor and the pathological events.

## Conclusion

This study provides novel insights into the pathological role of HSD10 overexpression and APP_Swe/Ind_-driven Aβ42 production in AD. We demonstrate for the first time that HSD10 overexpression alone compromises cell viability via mitochondrial dysfunction caused by elevated enzymatic activity, with associated impairments in the TCA cycle and β-oxidation. These findings highlight a distinct and independent pathological role of HSD10 in AD. In parallel, APP_Swe/Ind_ overexpression caused mitochondrial damage and triggered a shift toward glucose hypermetabolism, including upregulation of β-oxidation and alterations in the TCA cycle. These changes are consistent with a metabolic adaptation to compensate for the Aβ-induced cellular stress [49, 69]. Notably, both models exhibited increased vulnerability under glucose-free conditions, emphasizing the importance of impaired glucose utilization in AD pathology [89].

Moreover, we established the use of conditioned media from APP_Swe/Ind_ cells as a physiologically relevant source of Aβ42 for in vitro experiments. The cell-derived Aβ42 modulated HSD10 enzymatic activity and exacerbated HSD10-driven cellular damage. Importantly, this model enabled the evaluation of HSD10 inhibitors, effectively reducing observed pathology. Among them, the benzothiazole-based inhibitor **34** emerged as a promising therapeutic candidate, restoring cell viability and reducing cytotoxicity even under Aβ42-rich conditions.

## Electronic supplementary material

Below is the link to the electronic supplementary material.

Supplementary Material 1

## Data Availability

No datasets were generated or analysed during the current study.

## Abbreviations

Aβ: Amyloid-beta peptide
ACN: Acetonitrile
AD: Alzheimer’s disease
APP: Amyloid precursor protein
ATP: Adenosine triphosphate
C99: C-terminal 99 amino acid long fragment of amyloid precursor protein
CHANA: Cyclohexenyl amino naphthalene alcohol
CHANK: Cyclohexenyl amino naphthalene ketone
DMEM: Dulbecco’s Modified Eagle Medium
DMSO: Dimethyl sulfoxide
E2: 17β-estradiol
ECL: Excellent chemiluminescent substrate
ERAB: Endoplasmic reticulum-associated Aβ-binding protein; former name for HSD10
FA: Formic acid
FADH_2_/FAD: Flavin adenine dinucleotide
HEK293: Human embryonic kidney cells
HSD10: 17β-hydroxysteroid dehydrogenase type 10
LC-MS: Liquid chromatography-mass spectrometry
MAO: Monoamine oxidase
MEF: Mitochondrial electron flow
NADH/NAD^+^: Nicotinamide adenine dinucleotide
OXPHOS: Oxidative phosphorylation
PCR: Polymerase chain reaction
RCF: Relative centrifugal force
ROS: Reactive oxygen species
sAPPβ: N-terminal cleavage fragment of amyloid precursor protein
SD: Standard deviation
SDS-PAGE: Sodium dodecyl-sulphate polyacrylamide gel electrophoresis
SEM: Standard error of the mean
TCA: Tricarboxylic acid cycle
Wt: Wild-type

## References

[1] Yang SY, He XY, Isaacs C, Dobkin C, Miller D, Philipp M. Roles of 17β-hydroxysteroid dehydrogenase type 10 in neurodegenerative disorders. J Steroid Biochem Mol Biol. 2014;143:460–72. 25007702 10.1016/j.jsbmb.2014.07.001

[2] Holzmann J, Frank P, Löffler E, Bennett KL, Gerner C, Rossmanith W. RNase P without RNA: identification and functional reconstitution of the human mitochondrial tRNA processing enzyme. Cell. 2008;135(3):462–74. 18984158 10.1016/j.cell.2008.09.013

[3] Reinhard L, Sridhara S, Hallberg BM. Structure of the nuclease subunit of human mitochondrial RNase P. Nucleic Acids Res. 2015;43(11):5664–72. 25953853 10.1093/nar/gkv481 PMC4477676

[4] Reinhard L, Sridhara S, Hällberg BM. The MRPP1/MRPP2 complex is a tRNA-maturation platform in human mitochondria. Nucleic Acids Res. 2017;45(21):12469–80. 29040705 10.1093/nar/gkx902 PMC5716156

[5] Zschocke J, Ruiter JP, Brand J, Lindner M, Hoffmann GF, Wanders RJ, et al. Progressive infantile neurodegeneration caused by 2-methyl-3-hydroxybutyryl-CoA dehydrogenase deficiency: a novel inborn error of branched-chain fatty acid and isoleucine metabolism. Pediatr Res. 2000;48(6):852–5. 11102558 10.1203/00006450-200012000-00025

[6] Zschocke J. HSD10 disease: clinical consequences of mutations in the *HSD17B10* gene. J Inher Metab Disea. 2012;35(1):81–9. 10.1007/s10545-011-9415-4 22127393

[7] Rauschenberger K, Schöler K, Sass JO, Sauer S, Djuric Z, Rumig C, et al. A non-enzymatic function of 17β‐hydroxysteroid dehydrogenase type 10 is required for mitochondrial integrity and cell survival. EMBO Mol Med. 2010;2(2):51–62. 20077426 10.1002/emmm.200900055 PMC3377269

[8] Tieu K, Perier C, Vila M, Caspersen C, Zhang HP, Teismann P, et al. L-3-hydroxyacyl-CoA dehydrogenase II protects in a model of parkinson’s disease. Ann Neurol. 2004;56(1):51–60. 15236401 10.1002/ana.20133

[9] Du Yan S, Fu J, Soto C, Chen X, Zhu H, Al-Mohanna F, et al. An intracellular protein that binds amyloid-β peptide and mediates neurotoxicity in alzheimer’s disease. Nature. 1997;389(6652):689–95. 9338779 10.1038/39522

[10] Lustbader JW, Cirilli M, Lin C, Xu HW, Takuma K, Wang N, et al. ABAD directly links Aß to mitochondrial toxicity in alzheimer’s disease. Science. 2004;304(5669):448–52. 15087549 10.1126/science.1091230

[11] Yao J, Du H, Yan S, Fang F, Wang C, Lue LF, et al. Inhibition of Amyloid-β (Aβ) Peptide-Binding alcohol Dehydrogenase-Aβ interaction reduces Aβ accumulation and improves mitochondrial function in a mouse model of alzheimer’s disease. J Neurosci. 2011;31(6):2313–20. 21307267 10.1523/JNEUROSCI.4717-10.2011 PMC3381884

[12] Hamley IW. The amyloid Beta peptide: A chemist’s perspective. Role in alzheimer’s and fibrillization. Chem Rev. 2012;112(10):5147–92. 22813427 10.1021/cr3000994

[13] Shankar GM, Walsh DM. Alzheimer’s disease: synaptic dysfunction and Aβ. Mol Neurodegeneration. 2009;4(1):48. 10.1186/1750-1326-4-48 PMC2788538 19930651

[14] Chen XQ, Mobley WC. Alzheimer disease pathogenesis: insights from molecular and cellular biology studies of oligomeric Aβ and Tau species. Front Neurosci. 2019;13:659. 31293377 10.3389/fnins.2019.00659 PMC6598402

[15] Sun X, Chen WD, Wang YD. β-Amyloid: the key peptide in the pathogenesis of Alzheimer’s disease. Front Pharmacol [Internet]. 2015 Sep 30 [cited 2024 Oct 29];6. Available from: http://journal.frontiersin.org/Article/10.3389/fphar.2015.00221/abstract 10.3389/fphar.2015.00221 PMC4588032 26483691

[16] Zheng H, Koo EH. Biology and pathophysiology of the amyloid precursor protein. Mol Neurodegeneration. 2011;6(1):27. 10.1186/1750-1326-6-27 PMC3098799 21527012

[17] Small DH, Clarris HL, Williamson TG, Reed G, Key B, Mok SS, et al. Neurite-Outgrowth regulating functions of the amyloid protein precursor of alzheimer’s disease. J Alzheimer’s Disease. 1999;1(4–5):275–85. 12214125 10.3233/jad-1999-14-508

[18] Lorenzo A, Yuan M, Zhang Z, Paganetti PA, Sturchler-Pierrat C, Staufenbiel M, et al. Amyloid β interacts with the amyloid precursor protein: a potential toxic mechanism in alzheimer’s disease. Nat Neurosci. 2000;3(5):460–4. 10769385 10.1038/74833

[19] Ho A, Südhof TC. Binding of F-spondin to amyloid-β precursor protein: A candidate amyloid-β precursor protein ligand that modulates amyloid-β precursor protein cleavage. Proc Natl Acad Sci USA. 2004;101(8):2548–53. 14983046 10.1073/pnas.0308655100 PMC356987

[20] Lourenço FC, Galvan V, Fombonne J, Corset V, Llambi F, Müller U, et al. Netrin-1 interacts with amyloid precursor protein and regulates amyloid-β production. Cell Death Differ. 2009;16(5):655–63. 19148186 10.1038/cdd.2008.191 PMC2757418

[21] Young-Pearse TL, Bai J, Chang R, Zheng JB, LoTurco JJ, Selkoe DJ. A critical function for β-Amyloid precursor protein in neuronal migration revealed by *In utero* RNA interference. J Neurosci. 2007;27(52):14459–69. 18160654 10.1523/JNEUROSCI.4701-07.2007 PMC6673432

[22] Wang Z, Wang B, Yang L, Guo Q, Aithmitti N, Songyang Z, et al. Presynaptic and postsynaptic interaction of the amyloid precursor protein promotes peripheral and central synaptogenesis. J Neurosci. 2009;29(35):10788–801. 19726636 10.1523/JNEUROSCI.2132-09.2009 PMC2757256

[23] Oppermann U, Salim S, Hult M, Eissner G, Jörnvall H. Regulatory factors and motifs in SDR enzymes. In: Weiner H, Maser E, Crabb DW, Lindahl R, editors. Enzymology and molecular biology of carbonyl metabolism 7 [Internet]. Boston, MA: Springer US; 1999 [cited 2024 Jul 12]. pp. 365–71. (Advances in Experimental Medicine and Biology; vol. 463). Available from: http://link.springer.com/10.1007/978-1-4615-4735-8_45 10.1007/978-1-4615-4735-8_45 10352707

[24] Takuma K, Yao J, Huang J, Xu H, Chen X, Luddy J, et al. ABAD enhances Aβ-induced cell stress via mitochondrial dysfunction. FASEB J. 2005;19(6):1–25. 15665036 10.1096/fj.04-2582fje

[25] Yan SD, Stern DM. Mitochondrial dysfunction and alzheimer’s disease: role of amyloid-β peptide alcohol dehydrogenase (ABAD). Int J Exp Pathol. 2005;86(3):161–71. 15910550 10.1111/j.0959-9673.2005.00427.x PMC2517415

[26] Du Yan S, Shi Y, Zhu A, Fu J, Zhu H, Zhu Y, et al. Role of ERAB/l-3-Hydroxyacyl-coenzyme A dehydrogenase type II activity in Aβ-induced cytotoxicity. J Biol Chem. 1999;274(4):2145–56. 9890977 10.1074/jbc.274.4.2145

[27] Vinklarova L, Schmidt M, Benek O, Kuca K, Gunn-Moore F, Musilek K. Friend or enemy? Review of 17β‐HSD10 and its role in human health or disease. J Neurochem. 2020;155(3):231–49. 32306391 10.1111/jnc.15027

[28] Morsy A, Trippier PC. Amyloid-Binding alcohol dehydrogenase (ABAD) inhibitors for the treatment of alzheimer’s disease: miniperspective. J Med Chem. 2019;62(9):4252–64. 30444369 10.1021/acs.jmedchem.8b01530

[29] Benek O, Aitken L, Hroch L, Kuca K, Gunn-Moore F, Musilek K. A Direct Interaction Between Mitochondrial Proteins and Amyloid-β Peptide and its Significance for the Progression and Treatment of Alzheimer’s Disease. CMC. 2015;22(9):1056–85. 10.2174/0929867322666150114163051 25620098

[30] Benek O, Vaskova M, Miskerikova M, Schmidt M, Andrys R, Rotterova A, et al. Development of submicromolar 17β-HSD10 inhibitors and their in vitro and in vivo evaluation. Eur J Med Chem. 2023;258:115593. 37390508 10.1016/j.ejmech.2023.115593

[31] Schmidt M, Vaskova M, Rotterova A, Fiandova P, Miskerikova M, Zemanova L, et al. Physiologically relevant fluorescent assay for identification of 17β-hydroxysteroid dehydrogenase type 10 inhibitors. J Neurochem. 2023;167(2):154–67. 37458164 10.1111/jnc.15917

[32] Hanzlova M, Miskerikova MS, Rotterova A, Chalupova K, Jurkova K, Hamsikova M, et al. Nanomolar Benzothiazole-Based inhibitors of 17β-HSD10 with cellular bioactivity. ACS Med Chem Lett. 2023;14(12):1724–32. 38116418 10.1021/acsmedchemlett.3c00355 PMC10726454

[33] Xie Y, Deng S, Chen Z, Yan S, Landry DW. Identification of small-molecule inhibitors of the Aβ–ABAD interaction. Bioorg Med Chem Lett. 2006;16(17):4657–60. 16781151 10.1016/j.bmcl.2006.05.099

[34] Viswanath ANI, Kim T, Jung SY, Lim SM, Pae AN. In silico-designed novel non‐peptidic ABAD L_D_ hot spot mimetics reverse Aβ‐induced mitochondrial impairments in vitro. Chem Biol Drug Des. 2017;90(6):1041–55. 28660722 10.1111/cbdd.13065

[35] Morsy A, Maddeboina K, Gao J, Wang H, Valdez J, Dow LF, et al. Functionalized allopurinols targeting Amyloid-Binding alcohol dehydrogenase rescue Aβ-Induced mitochondrial dysfunction. ACS Chem Neurosci. 2022;13(14):2176–90. 35802826 10.1021/acschemneuro.2c00246

[36] Boutin S, Maltais R, Roy J, Poirier D. Synthesis of 17β-hydroxysteroid dehydrogenase type 10 steroidal inhibitors: selectivity, metabolic stability and enhanced potency. Eur J Med Chem. 2021;209:112909. 33081987 10.1016/j.ejmech.2020.112909

[37] Aitken L, Benek O, McKelvie BE, Hughes RE, Hroch L, Schmidt M, et al. Novel Benzothiazole-based Ureas as 17β-HSD10 inhibitors, A potential alzheimer’s disease treatment. Molecules. 2019;24(15):2757. 31362457 10.3390/molecules24152757 PMC6696238

[38] Kissinger CR, Rejto PA, Pelletier LA, Thomson JA, Showalter RE, Abreo MA, et al. Crystal structure of human ABAD/HSD10 with a bound inhibitor: implications for design of alzheimer’s disease therapeutics. J Mol Biol. 2004;342(3):943–52. 15342248 10.1016/j.jmb.2004.07.071

[39] Aitken B, McKelvie, Hughes, Hroch S, et al. Novel Benzothiazole-based Ureas as 17β-HSD10 inhibitors, A potential alzheimer’s disease treatment. Molecules. 2019;24(15):2757. 31362457 10.3390/molecules24152757 PMC6696238

[40] Abreo M, Meng J, Agree C. Pyrazole compounds, pharmaceutical compositions, and methods for modulating or inhibiting ERAB or HADH2 activity [Internet]. US20020065292A1, 2002 [cited 2025 Jun 17]. Available from: https://patents.google.com/patent/US20020065292A1/en

[41] Muirhead KEA, Froemming M, Li X, Musilek K, Conway SJ, Sames D, et al. (–)-CHANA, a fluorogenic probe for detecting amyloid binding alcohol dehydrogenase HSD10 activity in living cells. ACS Chem Biol. 2010;5(12):1105–14. 20836522 10.1021/cb100199m

[42] Shevchenko A, Tomas H, Havlis J, Olsen JV, Mann M. In-gel digestion for mass spectrometric characterization of proteins and proteomes. Nat Protoc. 2006;1(6):2856–60. 17406544 10.1038/nprot.2006.468

[43] Kong AT, Leprevost FV, Avtonomov DM, Mellacheruvu D, Nesvizhskii AI. MSFragger: ultrafast and comprehensive peptide identification in mass spectrometry-based proteomics. Nat Methods. 2017;14(5):513–20. 28394336 10.1038/nmeth.4256 PMC5409104

[44] Metodieva V, Smith T, Gunn-Moore F. The mitochondrial enzyme 17βHSD10 modulates ischemic and Amyloid-β-Induced stress in primary mouse astrocytes. eNeuro. 2022;9(5):ENEURO0040–222022. 10.1523/ENEURO.0040-22.2022 PMC9536859 36096650

[45] Tillement L, Lecanu L, Papadopoulos V. Further evidence on mitochondrial targeting of β-Amyloid and specificity of β-Amyloid-Induced mitotoxicity in neurons. Neurodegener Dis. 2011;8(5):331–44. 21311166 10.1159/000323264

[46] Caspersen C, Wang N, Yao J, Sosunov A, Chen X, Lustbader JW, et al. Mitochondrial Aβ: a potential focal point for neuronal metabolic dysfunction in alzheimer’s disease. FASEB J. 2005;19(14):2040–1. 16210396 10.1096/fj.05-3735fje

[47] Rossignol R, Gilkerson R, Aggeler R, Yamagata K, Remington SJ, Capaldi RA. Energy substrate modulates mitochondrial structure and oxidative capacity in Cancer cells. Cancer Res. 2004;64(3):985–93. 14871829 10.1158/0008-5472.can-03-1101

[48] Orlicka-Płocka M, Gurda-Wozna D, Fedoruk-Wyszomirska A, Wyszko E. Circumventing the Crabtree effect: forcing oxidative phosphorylation (OXPHOS) via galactose medium increases sensitivity of HepG2 cells to the purine derivative Kinetin riboside. Apoptosis. 2020;25(11–12):835–52. 32955614 10.1007/s10495-020-01637-x PMC7679298

[49] Atlante A, de Bari L, Bobba A, Amadoro G. A disease with a sweet tooth: exploring the Warburg effect in alzheimer’s disease. Biogerontology. 2017;18(3):301–19. 28314935 10.1007/s10522-017-9692-x

[50] Chen X, Qian Y, Wu S. The Warburg effect: evolving interpretations of an established concept. Free Radic Biol Med. 2015;79:253–63. 25277420 10.1016/j.freeradbiomed.2014.08.027 PMC4356994

[51] Traxler L, Herdy JR, Stefanoni D, Eichhorner S, Pelucchi S, Szücs A, et al. Warburg-like metabolic transformation underlies neuronal degeneration in sporadic alzheimer’s disease. Cell Metabol. 2022;34(9):1248–e12636. 10.1016/j.cmet.2022.07.014 PMC9458870 35987203

[52] Behl T, Kaur D, Sehgal A, Singh S, Sharma N, Zengin G, et al. Role of monoamine oxidase activity in alzheimer’s disease: an insight into the therapeutic potential of inhibitors. Molecules. 2021;26(12):3724. 34207264 10.3390/molecules26123724 PMC8234097

[53] Cai Z. Monoamine oxidase inhibitors: promising therapeutic agents for alzheimer’s disease (Review). Mol Med Rep. 2014;9(5):1533–41. 24626484 10.3892/mmr.2014.2040

[54] Emilsson L, Saetre P, Balciuniene J, Castensson A, Cairns N, Jazin EE. Increased monoamine oxidase messenger RNA expression levels in frontal cortex of alzheimer’s disease patients. Neurosci Lett. 2002;326(1):56–60. 12052537 10.1016/s0304-3940(02)00307-5

[55] Hemmerová E, Špringer T, Krištofiková Z, Homola J. Study of biomolecular interactions of mitochondrial proteins related to alzheimer’s disease: toward Multi-Interaction biomolecular processes. Biomolecules. 2020;10(9):1214. 32825572 10.3390/biom10091214 PMC7563123

[56] Reiss AB, Arain HA, Stecker MM, Siegart NM, Kasselman LJ. Amyloid toxicity in alzheimer’s disease. Rev Neurosci. 2018;29(6):613–27. 29447116 10.1515/revneuro-2017-0063

[57] Pera M, Larrea D, Guardia-Laguarta C, Montesinos J, Velasco KR, Agrawal RR, et al. Increased localization of APP ‐C99 in mitochondria‐associated ER membranes causes mitochondrial dysfunction in alzheimer disease. EMBO J. 2017;36(22):3356–71. 29018038 10.15252/embj.201796797 PMC5731665

[58] Chia CW, Egan JM, Ferrucci L. Age-related changes in glucose metabolism, hyperglycemia, and cardiovascular risk. Circ Res. 2018;123(7):886–904. 30355075 10.1161/CIRCRESAHA.118.312806 PMC6205735

[59] Kumar V, Kim SH, Bishayee K. Dysfunctional glucose metabolism in alzheimer’s disease onset and potential Pharmacological interventions. Int J Mol Sci. 2022;23(17):9540. 36076944 10.3390/ijms23179540 PMC9455726

[60] Maher AC, Akhtar M, Tarnopolsky MA. Men supplemented with 17β-estradiol have increased β-oxidation capacity in skeletal muscle. Physiol Genom. 2010;42(3):342–7. 10.1152/physiolgenomics.00016.2010 20484157

[61] Kitamura K, Erlangga JS, Tsukamoto S, Sakamoto Y, Mabashi-Asazuma H, Iida K. Daidzein promotes the expression of oxidative phosphorylation- and fatty acid oxidation-related genes via an estrogen-related receptor α pathway to decrease lipid accumulation in muscle cells. J Nutr Biochem. 2020;77:108315. 31923756 10.1016/j.jnutbio.2019.108315

[62] Toda K, Takeda K, Akira S, Saibara T, Okada T, Onishi S, et al. Alternations in hepatic expression of fatty-acid metabolizing enzymes in ArKO mice and their reversal by the treatment with 17β-estradiol or a peroxisome proliferator. J Steroid Biochem Mol Biol. 2001;79(1–5):11–7. 11850202 10.1016/s0960-0760(01)00135-2

[63] Houten SM, Wanders RJA. A general introduction to the biochemistry of mitochondrial fatty acid β-oxidation. J Inher Metab Disea. 2010;33(5):469–77. 10.1007/s10545-010-9061-2 PMC2950079 20195903

[64] Duran-Aniotz C, Hetz C. Glucose metabolism: A sweet relief of alzheimer’s disease. Curr Biol. 2016;26(17):R806–9. 27623263 10.1016/j.cub.2016.07.060

[65] Zhao J, Lang M. New insight into protein glycosylation in the development of alzheimer’s disease. Cell Death Discov. 2023;9(1):314. 37626031 10.1038/s41420-023-01617-5 PMC10457297

[66] Yan X, Hu Y, Wang B, Wang S, Zhang X. Metabolic dysregulation contributes to the progression of alzheimer’s disease. Front Neurosci. 2020;14:530219. 33250703 10.3389/fnins.2020.530219 PMC7674854

[67] Hipkiss AR, Aging. Alzheimer’s disease and dysfunctional glycolysis; similar effects of too much and too little. Aging Disease. 2019;10(6):1328. 31788344 10.14336/AD.2019.0611 PMC6844594

[68] Bell SM, Burgess T, Lee J, Blackburn DJ, Allen SP, Mortiboys H. Peripheral Glycolysis in neurodegenerative diseases. IJMS. 2020;21(23):8924. 33255513 10.3390/ijms21238924 PMC7727792

[69] Bobba A, Atlante A, Azzariti A, Sgaramella G, Calissano P, Marra E. Mitochondrial impairment induces excitotoxic death in cerebellar granule cells. Int J Mol Med [Internet]. 2004 Jun 1 [cited 2024 Oct 11]; Available from: http://www.spandidos-publications.com/10.3892/ijmm.13.6.873 15138628

[70] Zhelev Z, Sumiyoshi A, Aoki I, Lazarova D, Vlaykova T, Higashi T, et al. Over-Reduced state of mitochondria as a trigger of β-Oxidation shuttle in Cancer cells. Cancers (Basel). 2022;14(4):871. 35205619 10.3390/cancers14040871 PMC8870273

[71] Lee H, Woo SM, Jang H, Kang M, Kim SY. Cancer depends on fatty acids for ATP production: A possible link between cancer and obesity. Sem Cancer Biol. 2022;86:347–57. 10.1016/j.semcancer.2022.07.005 35868515

[72] Reed MN, Hofmeister JJ, Jungbauer L, Welzel AT, Yu C, Sherman MA, et al. Cognitive effects of cell-derived and synthetically derived Aβ oligomers. Neurobiol Aging. 2011;32(10):1784–94. 20031278 10.1016/j.neurobiolaging.2009.11.007 PMC2895944

[73] Ono K, Condron MM, Teplow DB. Structure–neurotoxicity relationships of amyloid β-protein oligomers. Proc Natl Acad Sci USA. 2009;106(35):14745–50. 19706468 10.1073/pnas.0905127106 PMC2736424

[74] Oppermann UCT, Salim S, Tjernberg LO, Terenius L, Jörnvall H. Binding of amyloid β-peptide to mitochondrial hydroxyacyl-CoA dehydrogenase (ERAB): regulation of an SDR enzyme activity with implications for apoptosis in alzheimer’s disease. FEBS Lett. 1999;451(3):238–42. 10371197 10.1016/s0014-5793(99)00586-4

[75] Powell AJ, Read JA, Banfield MJ, Gunn-Moore F, Yan SD, Lustbader J, et al. Recognition of structurally diverse substrates by type II 3-hydroxyacyl-CoA dehydrogenase (HADH II)/Amyloid-β binding alcohol dehydrogenase (ABAD)1. J Mol Biol. 2000;303(2):311–27. 11023795 10.1006/jmbi.2000.4139

[76] Yan Y, Liu Y, Sorci M, Belfort G, Lustbader JW, Yan SS, et al. Surface plasmon resonance and nuclear magnetic resonance studies of ABAD-Abeta interaction. Biochemistry. 2007;46(7):1724–31. 17253767 10.1021/bi061314n

[77] Mrdenovic D, Lipkowski J, Pieta P. Analyzing morphological properties of early-stage toxic amyloid β oligomers by atomic force microscopy. In: Cranfield CG, editor. Membrane lipids [Internet]. New York, NY: Springer US; 2022 [cited 2024 Oct 2]. 227–41. (Methods in Molecular Biology; vol. 2402). Available from: https://link.springer.com/10.1007/978-1-0716-1843-1_18 10.1007/978-1-0716-1843-1_18 34854048

[78] García-Ayllón MS, Lopez-Font I, Boix CP, Fortea J, Sánchez-Valle R, Lleó A, et al. C-terminal fragments of the amyloid precursor protein in cerebrospinal fluid as potential biomarkers for alzheimer disease. Sci Rep. 2017;7(1):2477. 28559572 10.1038/s41598-017-02841-7 PMC5449401

[79] Podlisny MB, Mammen AL, Schlossmacher MG, Palmert MR, Younkin SG, Selkoe DJ. Detection of soluble forms of the β-amyloid precursor protein in human plasma. Biochem Biophys Res Commun. 1990;167(3):1094–101. 2138892 10.1016/0006-291x(90)90635-z

[80] Rose C, Peoc’h K, Chasseigneaux S, Paquet C, Dumurgier J, Bourasset F, et al. New highly sensitive rodent and human tests for soluble amyloid precursor protein alpha quantification: preclinical and clinical applications in alzheimer’s disease. BMC Neurosci. 2012;13(1):84. 22824057 10.1186/1471-2202-13-84 PMC3418197

[81] Araki W, Hattori K, Kanemaru K, Yokoi Y, Omachi Y, Takano H, et al. Re-evaluation of soluble APP-α and APP-β in cerebrospinal fluid as potential biomarkers for early diagnosis of dementia disorders. Biomark Res. 2017;5(1):28. 29018524 10.1186/s40364-017-0108-5 PMC5610422

[82] Kang JH, Korecka M, Toledo JB, Trojanowski JQ, Shaw LM. Clinical utility and analytical challenges in measurement of cerebrospinal fluid Amyloid-β1–42 and τ proteins as alzheimer disease biomarkers. Clin Chem. 2013;59(6):903–16. 23519967 10.1373/clinchem.2013.202937 PMC4159709

[83] Shigemizu D, Asanomi Y, Akiyama S, Mitsumori R, Niida S, Ozaki K. Whole-genome sequencing reveals novel ethnicity-specific rare variants associated with alzheimer’s disease. Mol Psychiatry. 2022;27(5):2554–62. 35264725 10.1038/s41380-022-01483-0 PMC9135624

[84] Hernandez-Rapp J, Rainone S, Goupil C, Dorval V, Smith PY, Saint-Pierre M, et al. microRNA-132/212 deficiency enhances Aβ production and senile plaque deposition in alzheimer’s disease triple Transgenic mice. Sci Rep. 2016;6(1):30953. 27484949 10.1038/srep30953 PMC4971468

[85] Leal NS, Schreiner B, Pinho CM, Filadi R, Wiehager B, Karlström H, et al. Mitofusin-2 knockdown increases ER–mitochondria contact and decreases amyloid β‐peptide production. J Cell Mol Medi. 2016;20(9):1686–95. 10.1111/jcmm.12863 PMC4988279 27203684

[86] Khan I, Krishnaswamy S, Sabale M, Groth D, Wijaya L, Morici M, et al. Efficient production of a mature and functional gamma secretase protease. Sci Rep. 2018;8(1):12834. 30150752 10.1038/s41598-018-30788-w PMC6110731

[87] Shaw G, Morse S, Ararat M, Graham FL. Preferential transformation of human neuronal cells by human adenoviruses and the origin of HEK 293 cells. FASEB J. 2002;16(8):869–71. 11967234 10.1096/fj.01-0995fje

[88] Thomas P, Smart TG. HEK293 cell line: A vehicle for the expression of Recombinant proteins. J Pharmacol Toxicol Methods. 2005;51(3):187–200. 15862464 10.1016/j.vascn.2004.08.014

[89] Daulatzai MA. Cerebral hypoperfusion and glucose hypometabolism: key pathophysiological modulators promote neurodegeneration, cognitive impairment, and alzheimer’s disease. J Neurosci Res. 2017;95(4):943–72. 27350397 10.1002/jnr.23777

