# Supplementary material for: New insights into the 17β-hydroxysteroid dehydrogenase type 10 and amyloid-β 42 derived cytotoxicity relevant to Alzheimer’s disease
Source: Alzheimers Res Ther. 2025 Jul 23;17:170. doi: 10.1186/s13195-025-01821-8 (PMC12285154; doi:10.1186/s13195-025-01821-8)
Supplement: Supplementary file 1 — Supplementary Material 1 [file 13195_2025_1821_MOESM1_ESM.docx]

*SUPPLEMENTARY INFORMATION*

**New insights into the 17β-hydroxysteroid dehydrogenase type 10 and amyloid-β 42 derived cytotoxicity relevant to Alzheimer’s disease**

Aneta Houfková^1^; Monika Schmidt^1,2^*; Ondřej Benek^1^; Ivo Fabrik^2^; Rudolf Andrýs^1^; Lucie Zemanová^1^; Ondřej Soukup^2^ and Kamil Musílek^1^

^1^ *University of Hradec Kralove, Faculty of Science, Department of Chemistry, Rokitanskeho 62, 500 03 Hradec Kralove, Czech Republic*

^2^ *University Hospital Hradec Kralove, Biomedical Research Centre, Sokolska 581, 500 03 Hradec Kralove, Czech Republic*

*****Correspondence: [monika.schmidt@fnhk.cz](mailto:monika.schmidt@fnhk.cz)

**TABLE OF CONTENTS:**

1. METHODS
   1. Extracellular oxygen consumption rate analyses
   2. Cell viability determination using resazurin
2. RESULTS
   1. Appendix Figure S1: Immunoblotting analysis of HSD10, and HSD10_mut_ cell (related to Figure 2A).
   2. Appendix Figure S2: Immunoblotting analysis of HSD10, and HSD10_mut_ cell (related to Figure 2A).
   3. Appendix Table S3: HSD10 activity determination in HEK293_wt_, HSD10, HSD10_mut_, and APP_Swe/Ind_ cells (related to Figures 2B and 3D).
   4. Appendix Figure S4: Immunoblotting analysis of APP_Swe/Ind_ cells (related to Figure 3A).
   5. Appendix Figure S5: Immunoblotting analysis of APP_Swe/Ind_ cells (related to Figure 3A).
   6. Appendix Figure S6: Immunoblotting analysis of APP_Swe/Ind_ cells (related to Figure 3B).
   7. Appendix Figure S7: Immunoblotting analysis of APP_Swe/Ind_ conditioned medium (related to Figure 3C).
   8. Appendix Figure S8: Immunoblotting analysis of APP_Swe/Ind_ conditioned medium (related to Figure 3C).
   9. Appendix Figure S9: Analysis of APP_Swe/Ind_ conditioned medium (related to Figures 4A and 4B).
   10. Appendix Figure S10: Immunoblotting analysis of APP_Swe/Ind_ conditioned medium fractions (related to Figure 4C).
   11. Appendix Table S11: Chromatogram of standards’ separation by size-exclusion chromatography (related to Figure 4C).
   12. Appendix Table S12: ATP levels and cytotoxicity in HEK293_wt_, HSD10, HSD10_mut_, and APP_Swe/Ind_ cells (related to Figures 5A and 5B).
   13. Appendix Table S13: Viability of HEK293_wt_, HSD10, HSD10_mut_, and APP_Swe/Ind_ cells (related to Figures 5C).
   14. Appendix Table S14: Mitochondrial toxicity of HEK293, HSD10, and APP_Swe/Ind_ cells (related to Figures 5D).
   15. Appendix Figure S15: Mitochondrial electron flow changes (corresponding to metabolic changes) in HEK293_wt_, HSD10, and APP_Swe/Ind_ cells (related to Figure 6).
   16. Appendix Table S16: ATP levels and cytotoxicity in HEK293_wt_, and HSD10 cells after cell-produced Aβ42 treatment (related to Figure 7C).
   17. Appendix Table S17: ATP levels and cytotoxicity in HSD10 cells after HSD10-inhibitors treatment (related to Figure 9A).
   18. Appendix Table S18: ATP levels and cytotoxicity in HSD10 cells after HSD10-inhibitors and cell-produced Aβ42 treatment (related to Figure 9B).
   19. Appendix Table S19: Mitochondrial electron flow measurements (corresponding to metabolic changes) in HEK293_wt_, HSD10, and APP_Swe/Ind_ cells (related to Figure 6 and S15).
   20. Appendix Figure S20: Oxygen consumption rates in HSD10 cells and HEK293_wt_ cells (additional method to Figure 6C and S15B).
   21. Appendix Figure S21: Viability of HSD10 cells (determined using the resazurin assay) after HSD10-inhibitors and cell-produced Aβ42 treatment (additional method to Figure 9B).

**METHODS:**

**Extracellular oxygen consumption rate analyses**

To confirm the changes in mitochondrial functions between the APP_Swe/Ind_ and HSD10 cells, the determination of basal respiration using the extracellular oxygen consumption kit (Abcam, ab197243) was performed. Before the measurement, the cells were cultivated for 48 hr in complete galactose media. The cells (0.1x10^6^ in galactose media) were seeded into a 96-black well clear-bottom plate (Brand, BR781971) immediately before the measurement, treated by 1µM antimycin A (control cells), the extracellular O_2_ consumption reagent (probe) was added and each well was overloaded by high sensitivity mineral oil to limit the back diffusion of ambient oxygen. The fluorescence measurements using the time-resolved fluorescence were set in two integration windows as follows: excitation wavelength: 380 ± 20 nm, emission wavelength: 650 ± 20 nm; integration window 1: 30 µs delay (D1), 30 µs measurement time (W1); integration window 2: 70 µs delay (D2), 30 µs measurement time (W2). The reaction was measured kinetically on a Tecan Spark 10 M instrument at 1-min intervals for a total of 3 hr after seeding. The dual intensity readings were used to calculate the corresponding Lifetime using the following transformation: Lifetime (µs) [T] = (D2-D1)/ln(W1/W2).

**Cell viability determination using resazurin**

To verify the potential of selected inhibitors to reverse pathology associated with HSD10 overexpression in an Aβ42-rich environment, an additional viability assay using the Resazurin Assay Kit (Abcam, ab129732) was performed. For this purpose, 1x10⁴ cells were seeded in 100 µL of either galactose culture medium or Aβ42-conditioned galactose medium (APP_Swe/Ind_ cells conditioned galactose medium) per well into a black 96-well microplate with a clear bottom (Brand, BR781971), together with the respective inhibitor treatments. Cells were cultured for 72 hr.

The assay readout was performed using the TECAN Spark 10 M instrument following the manufacturer’s protocol with minor modifications. A 20x concentrated resazurin stock solution was diluted to a 5x working concentration using a fresh galactose culture medium. Subsequently, 10 µL of this 5x resazurin solution was added to each well containing 100 µL of culture medium with cells. Following reagent addition, cells were incubated for an additional 4 hr at 37 °C, and fluorescence was measured at Ex/Em = 550/590 nm.

**RESULTS:**

**
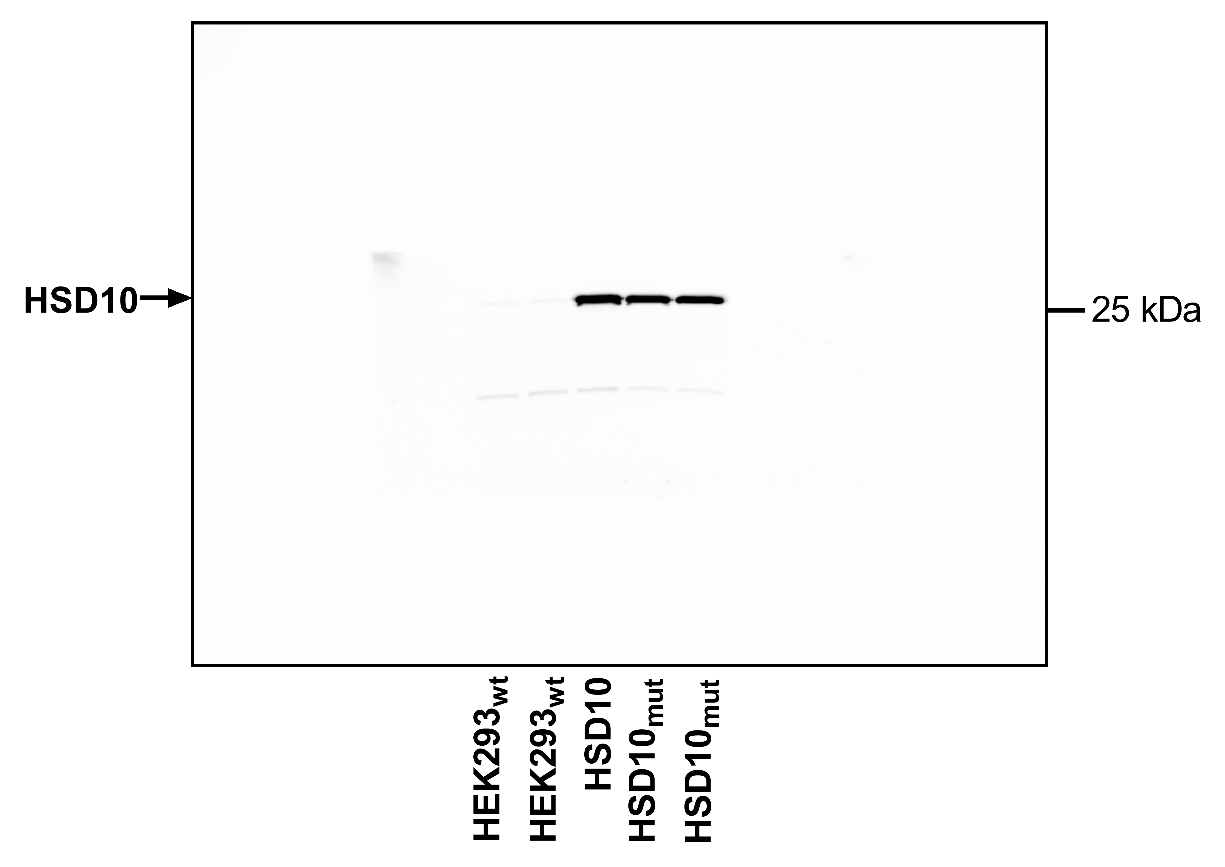
Figure S1**: Original western blot image from HEK293_wt_, HSD10, and HSD10_mut_ cell lysates immunoblotting analysis using an anti-HSD10 primary antibody.

**
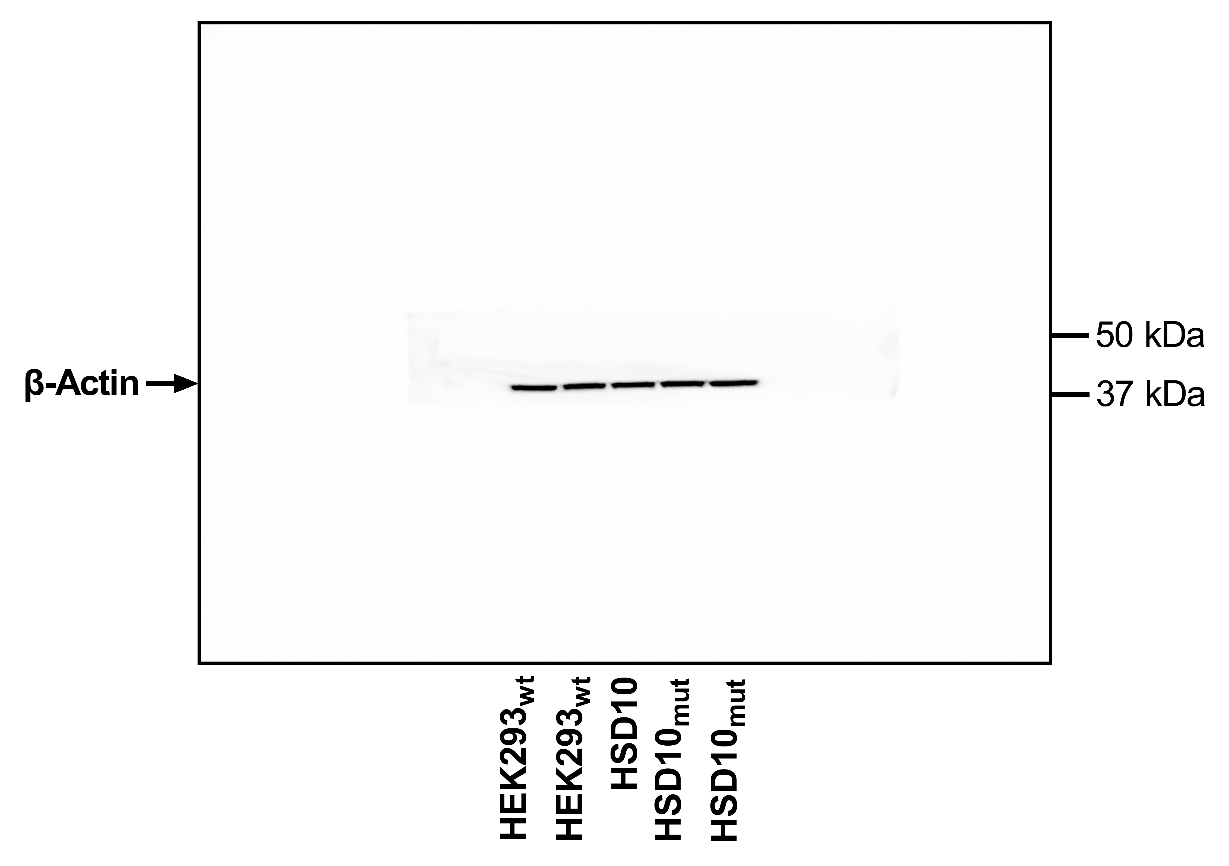
Figure S2:** Original western blot image from HEK293_wt_, HSD10, and HSD10_mut_ cell lysates immunoblotting analysis using an anti-β-Actin primary antibody.

**Table S3:** HSD10 activity determination via (-)-CHANA to CHANK turnover in HEK293_wt_, HSD10, HSD10_mut_, and APP_Swe/Ind_ cells.

| **Cell line** |  | **ΔF (2 hr)** |
| --- | --- | --- |
| **HEK293_wt_** |  | 7364.23 ± 83.61 |
| **HSD10** |  | 8561.54 ± 73.71 |
| **HSD10_mut_** |  | 7405.74 ± 79.48 |
| **APP_Swe/Ind_** |  | 7062.18 ± 95.52 |

**
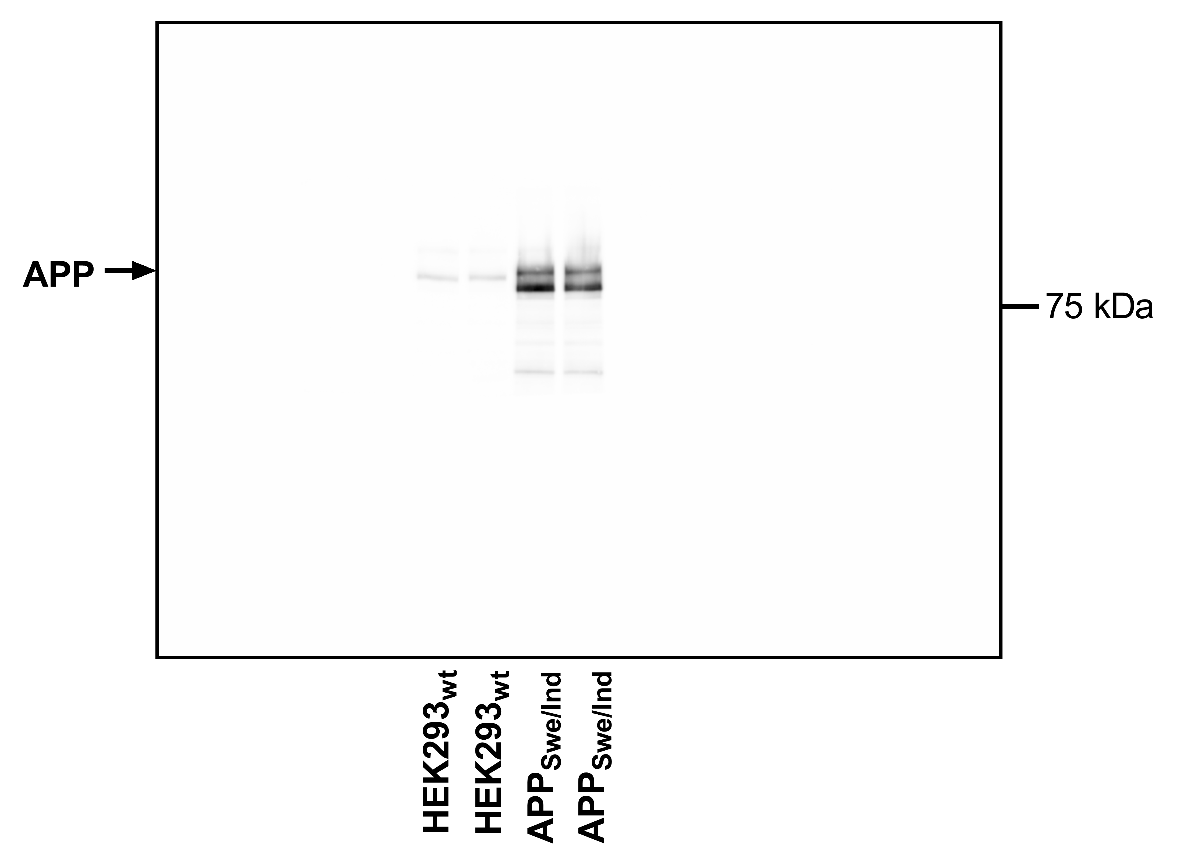
Figure S4**: Original western blot image from HEK293_wt_ and APP_Swe/Ind_ cell lysates immunoblotting analysis using an anti-APP primary antibody.

**
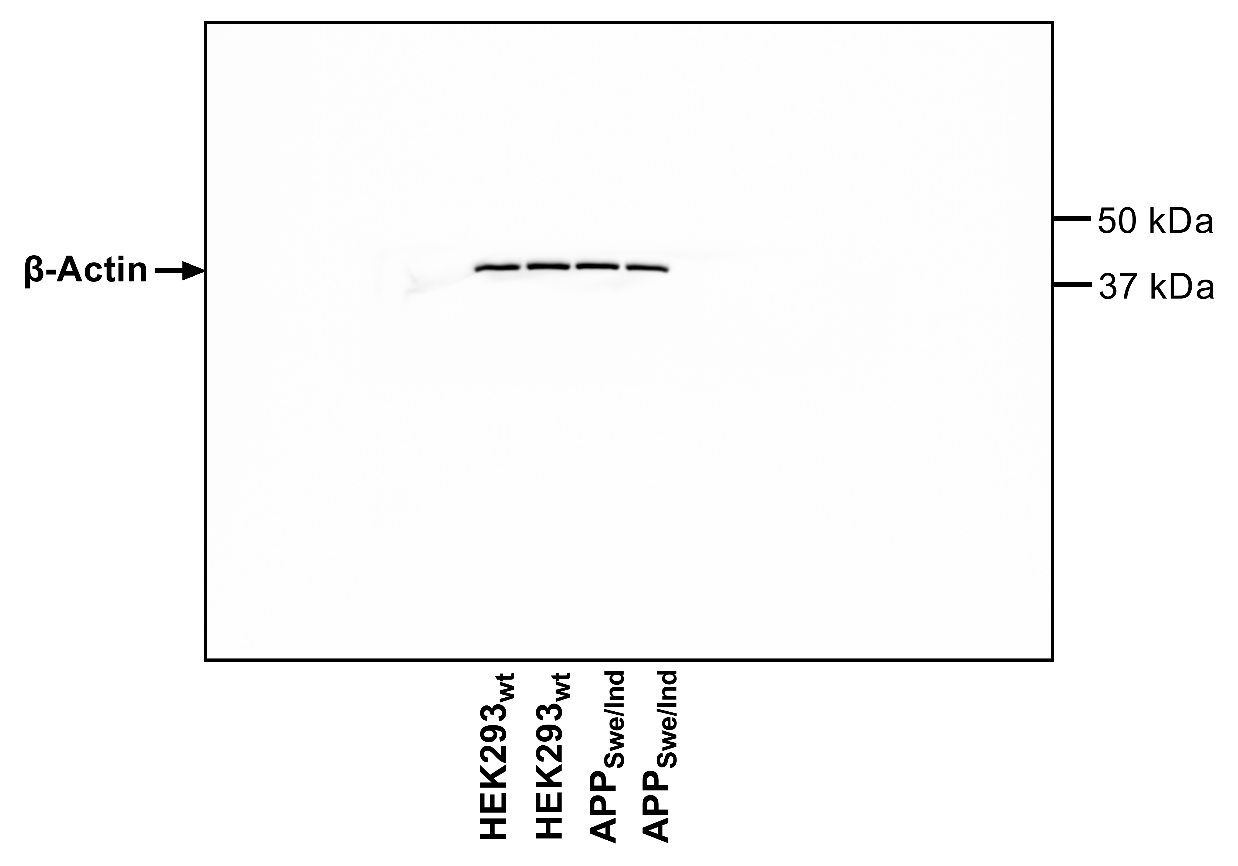
Figure S5:** Original western blot image from HEK293_wt_ and APP_Swe/Ind_ cell lysates immunoblotting analysis using an anti-β-Actin primary antibody.


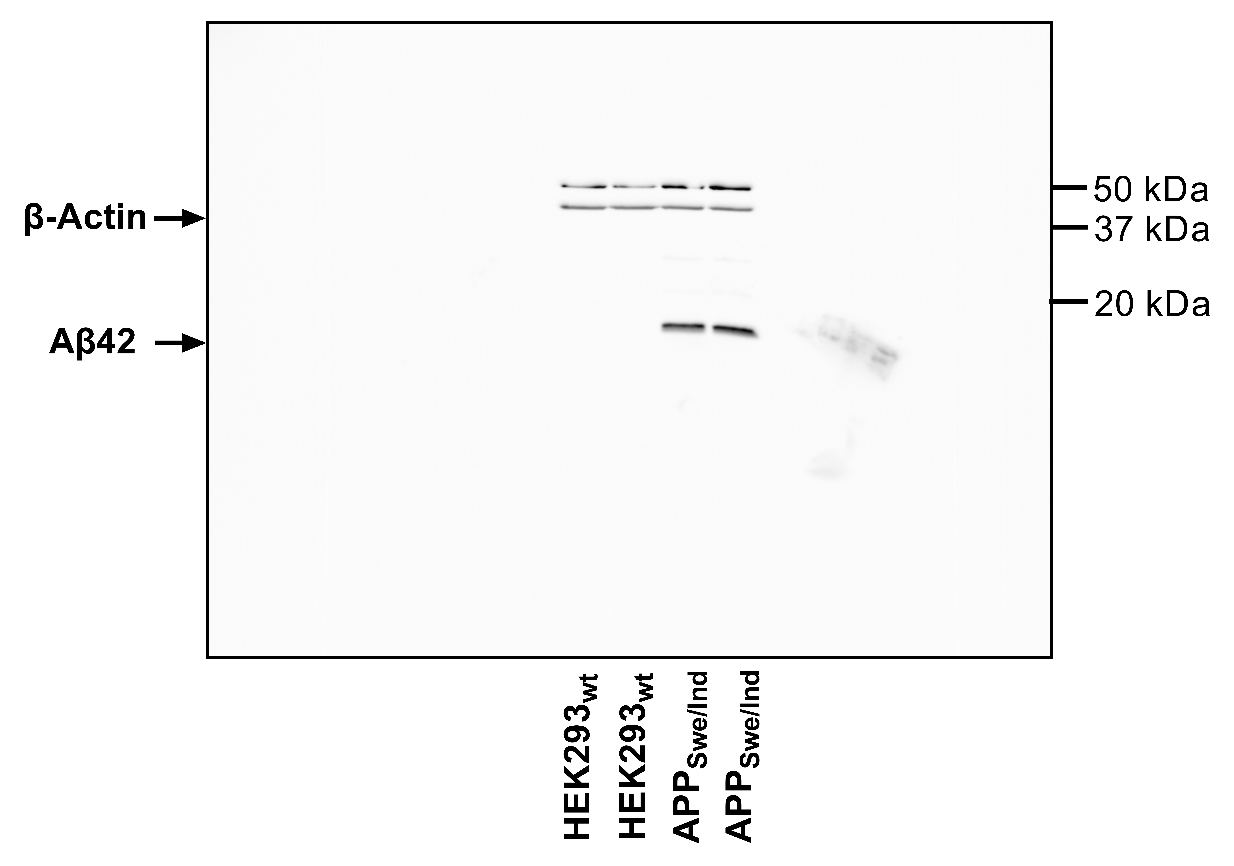
**Figure S6:** Original western blot image from HEK293_wt_ and APP_Swe/Ind_ cell lysates immunoblotting analysis using a combination of anti-Aβ, and anti-β-actin primary antibodies.

**
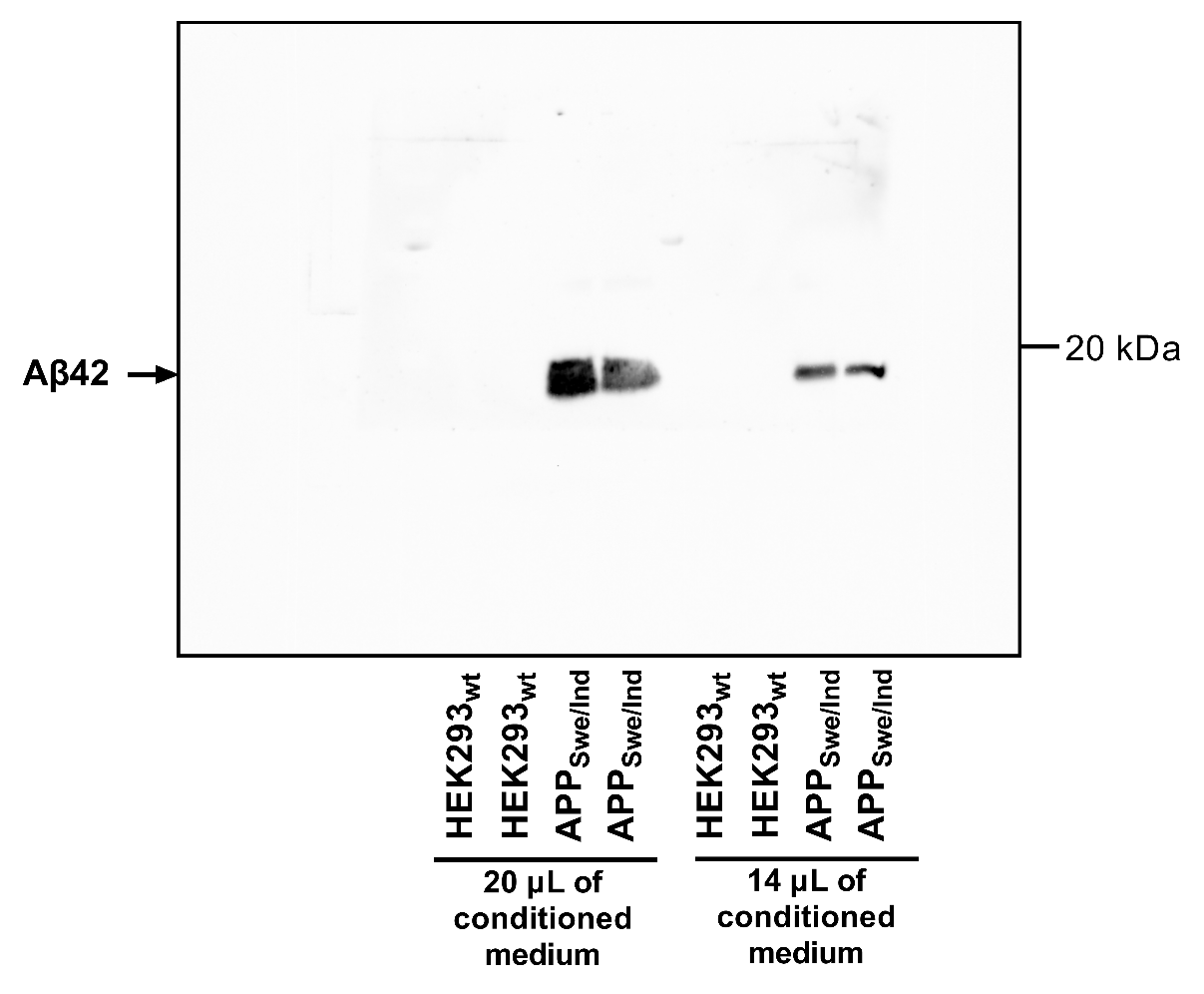
Figure S7:** Original western blot image from HEK293_wt_ and APP_Swe/Ind_ cell conditioned medium immunoblotting analysis using an anti-Aβ primary antibody.

**
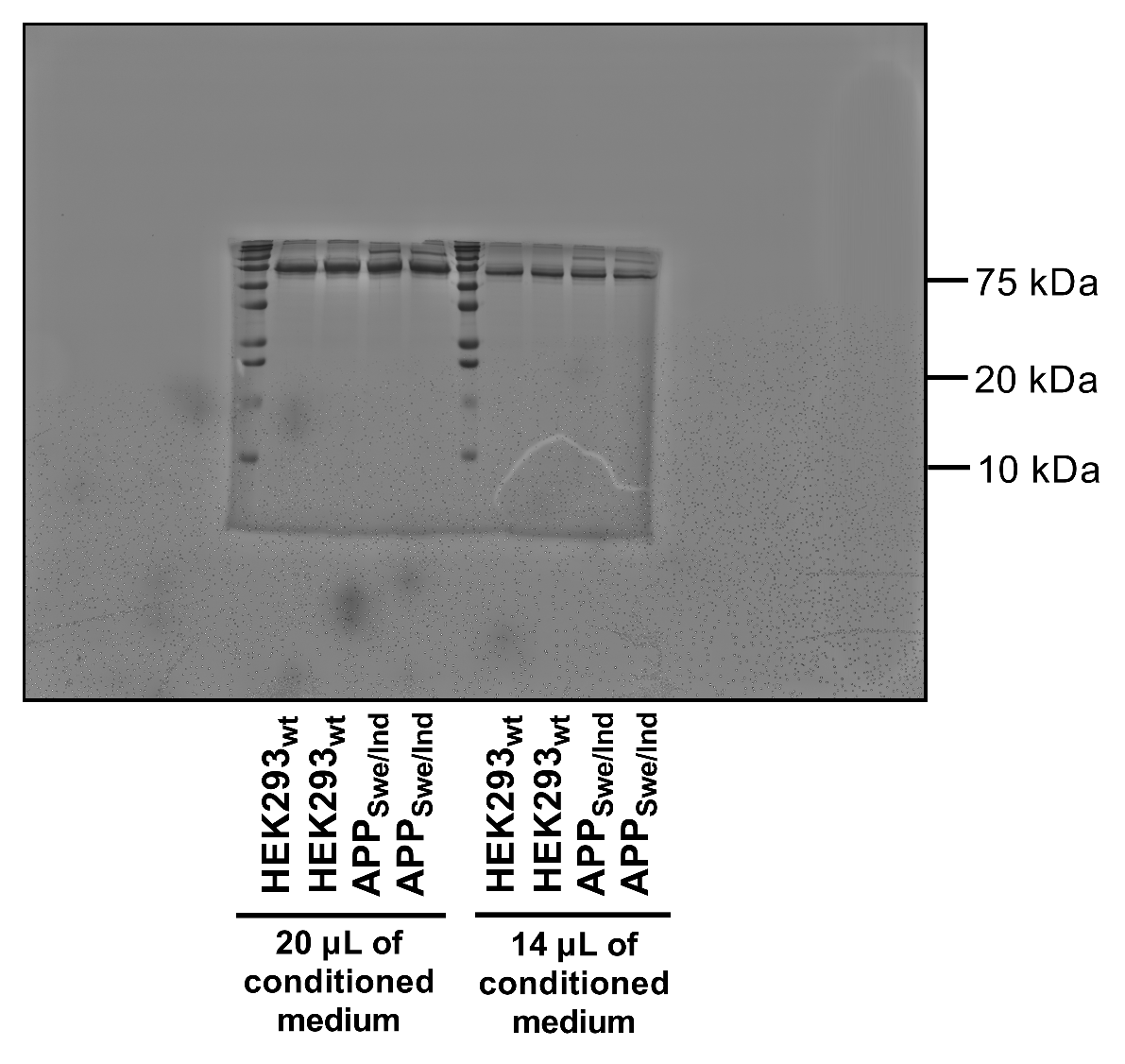
Figure S8:** Original gel staining image from HEK293_wt_ and APP_Swe/Ind_ cells conditioned medium immunoblotting analysis.


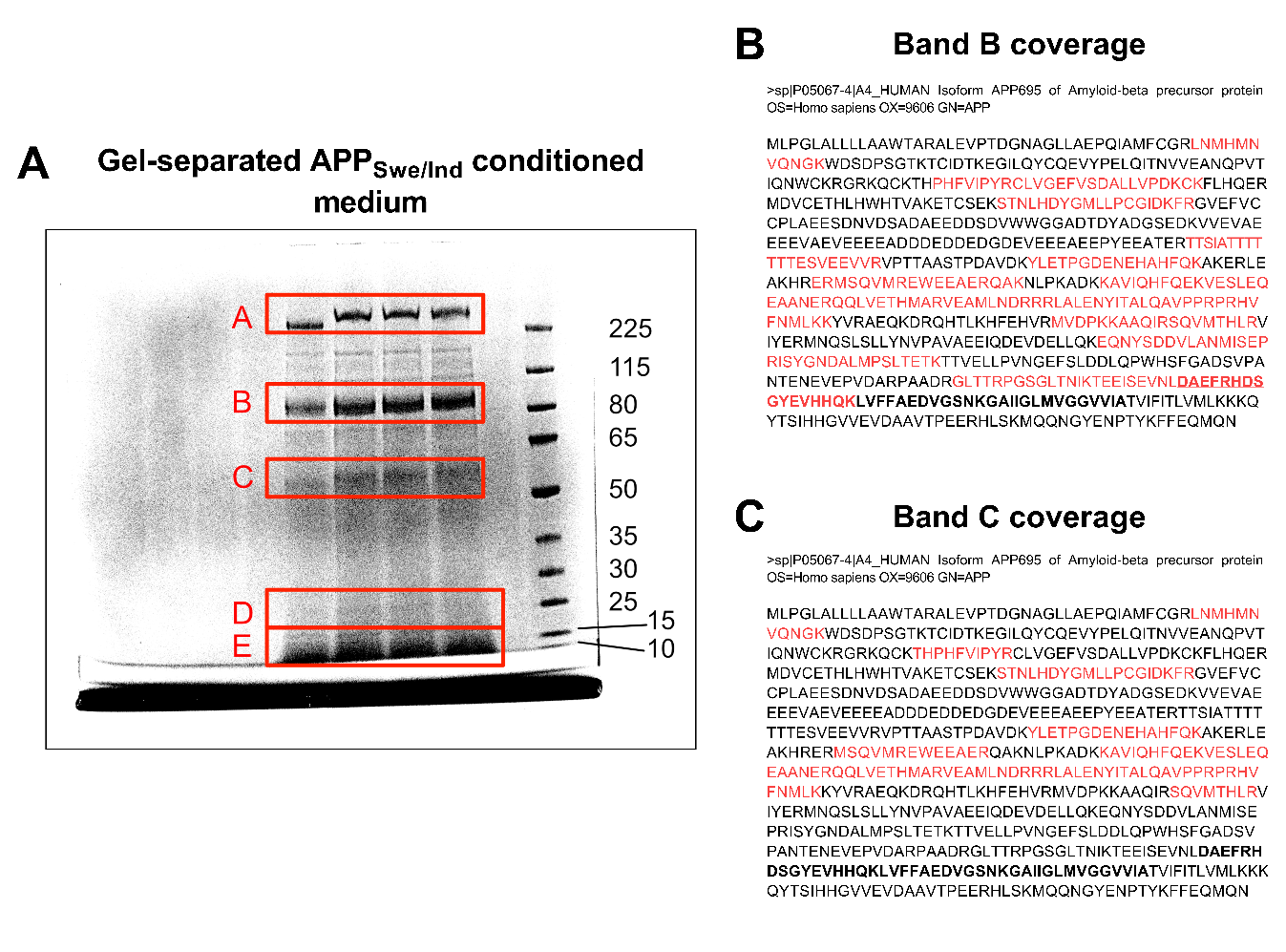
**Figure S9:** Analysis of APP_Swe/Ind_ conditioned medium. (**A**) The APP_Swe/Ind_ conditioned serum and protein-free medium was desalted, and column separated, individual fractions were lyophilized, and gel-separated in four lanes of NuPAGE gel. Visible bands from the concentrated samples were excised and subjected to mass-spectrometric analysis, which revealed the presence of APP or APP-derived fragments in Band B, C, and E. (**B**) Coverage (marked in red) of tryptically digested proteins from Band B with the FASTA sequence of APP_Swe/Ind_. The results suggest the presence of full-length APP_Swe/Ind_ protein. (**C**) Coverage (marked in red) of tryptically digested proteins from Band C with the FASTA sequence of APP_Swe/Ind_. The results suggest the presence of APP-derived N-terminal cleavage fragments.

**
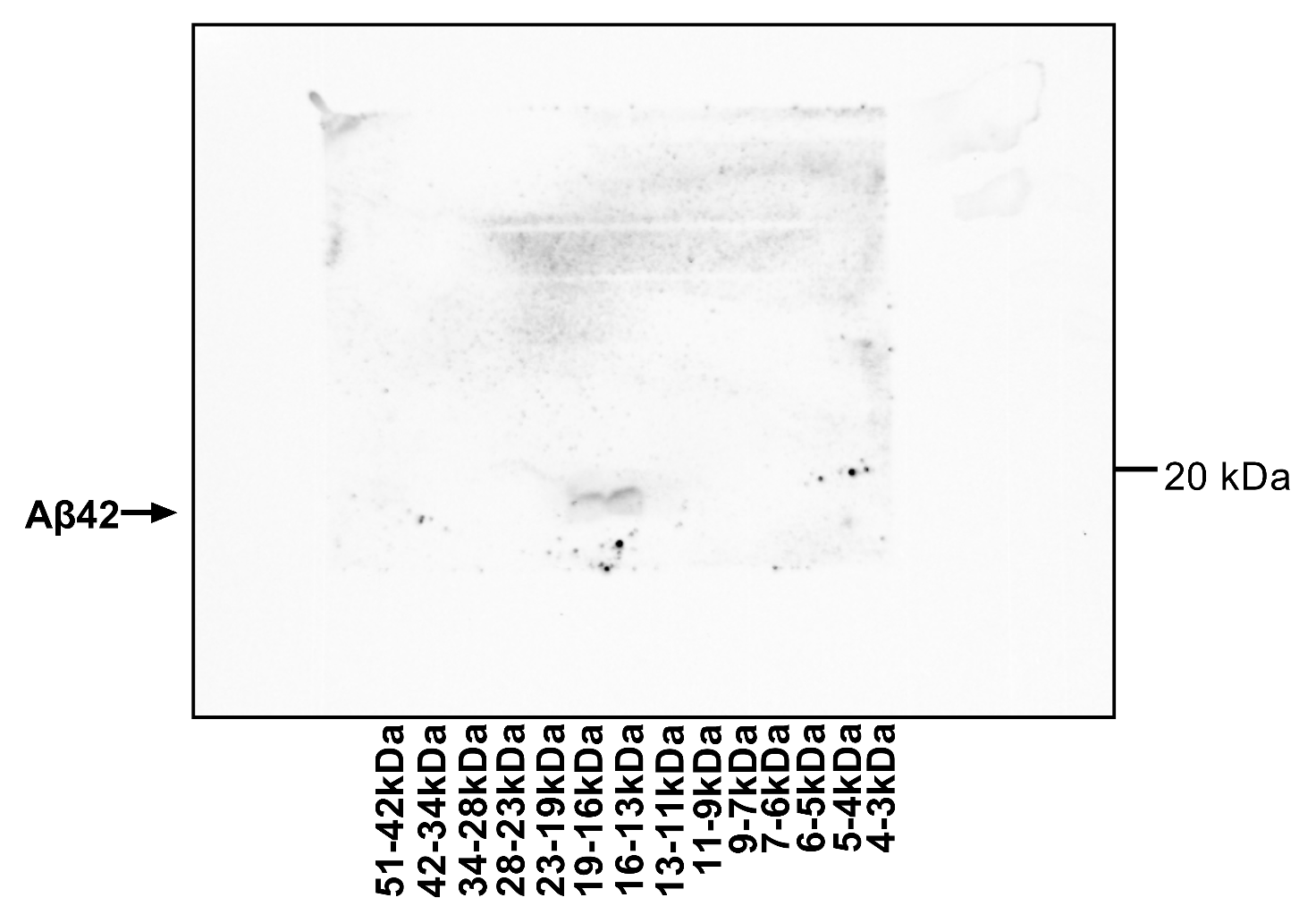
Figure S10:** Original western blot image from APP_Swe/Ind_ conditioned medium fractions immunoblotting analysis using an anti-Aβ primary antibody.


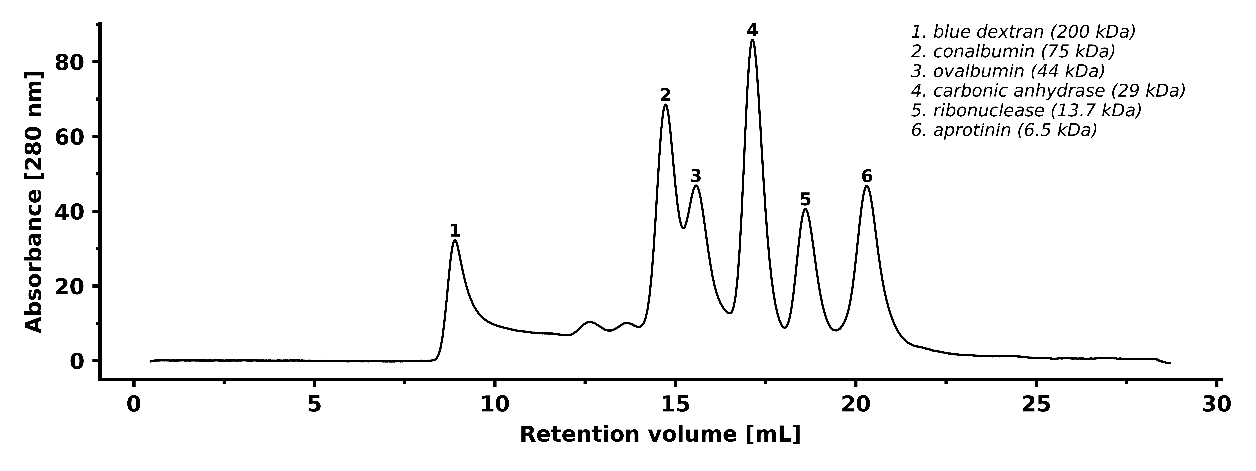


**Figure S11:** Size-exclusion chromatogram of the Cytiva low molecular weight gel filtration calibration kit (28403841) using a Superdex 75 10/300 GL column. Mobile phase: PBS, flow rate: 0.7 mL/min, detection at 280 nm. Peaks are labeled with their corresponding molecular weights (kDa).

**Table S12:** ATP levels and cytotoxicity in HEK293_wt_, HSD10, HSD10_mut_, and APP_Swe/Ind_ cells 72 hr and 168 hr post-seeding into the glucose and galactose medium. Data were normalized between DMSO-treated (1%) and valinomycin-treated (100 µM) HEK293_wt_ cells cultivated in glucose media. Values are given as means ± SD from three independent cell culture preparations with four technical replicates (n=12). SD; standard deviation.

| **Cell line** |  | **72 hr** | | | | |  | **168 hr** | | | | |
| --- | --- | --- | --- | --- | --- | --- | --- | --- | --- | --- | --- | --- |
|  |  | **Glucose** | |  | **Galactose** | |  | **Glucose** | |  | **Galactose** | |
|  |  | **% of ATP quantity** | **% of Cytotoxicity** |  | **% of ATP quantity** | **% of Cytotoxicity** |  | **% of ATP quantity** | **% of Cytotoxicity** |  | **% of ATP quantity** | **% of Cytotoxicity** |
| **HEK293_wt_** |  | 100.00 ± 2.33 | 0.00 ± 1.65 |  | 89.45 ± 3.84 | 2.86 ± 2.61 |  | 100.00 ± 2.52 | 0.00 ± 1.45 |  | 87.05 ± 3.93 | 4.17 ± 2.70 |
| **HSD10** |  | 76.67 ± 3.09 | 1.81 ± 1.67 |  | 69.23 ± 2.78 | 14.63 ± 3.09 |  | 71.75 ± 6.53 | 4.52 ± 3.64 |  | 50.11 ± 4.42 | 39.33 ± 5.09 |
| **HSD10_mut_** |  | 93.98 ± 8.66 | 1.64 ± 0.78 |  | 84.49 ± 4.82 | 3.76 ± 2.33 |  | 92.07 ± 4.29 | 3.36 ± 1.07 |  | 82.46 ± 4.12 | 5.58 ± 1.45 |
| **APP_Swe/Ind_** |  | 66.37 ± 3.98 | 3.06 ± 2.53 |  | 54.05 ± 4.35 | 11.33 ± 2.25 |  | 63.18 ± 5.19 | 5.33 ± 3.60 |  | 46.82 ± 4.35 | 37.73 ± 3.37 |

**Table S13:** Viability of HEK293_wt_, HSD10, HSD10_mut_, and APP_Swe/Ind_ cells monitored for 72 hr of galactose media cultivation. Values are given as means ± SD from three independent cell culture preparations with three technical replicates (n=9). SD; standard deviation.

| **Cell line** | **Viability (RLU)** | | | | | | | | | |
| --- | --- | --- | --- | --- | --- | --- | --- | --- | --- | --- |
|  | **0 hr** | **6 hr** | **12 hr** | **24 hr** | **30 hr** | **36 hr** | **48 hr** | **54 hr** | **60 hr** | **72 hr** |
| **HEK293_wt_** | 12174 ± 1179 | 27338 ± 1684 | 40672 ± 3151 | 59211 ± 4932 | 67956 ± 2926 | 75202 ± 2529 | 86520 ± 1828 | 93972 ± 2230 | 104795 ± 3664 | 121752 ± 6226 |
| **HSD10** | 9733 ± 303 | 22567 ± 899 | 33489 ± 1151 | 52152 ± 1950 | 57042 ± 2181 | 63138 ± 2641 | 67950 ± 2408 | 71303 ± 3146 | 75670 ± 1877 | 82197 ± 4629 |
| **HSD10_mut_** | 12057 ± 988 | 23385 ± 2085 | 36381 ± 1996 | 52922 ± 4330 | 60904 ± 4668 | 70349 ± 2816 | 79650 ± 3233 | 87653 ± 2744 | 98823 ± 3299 | 109114 ± 2935 |
| **APP_Swe/Ind_** | 7877 ± 960 | 20243 ± 721 | 28929 ± 1043 | 44196 ± 1847 | 48473 ± 2210 | 55747 ± 2857 | 64082 ± 3632 | 70102 ± 3555 | 76341 ± 3276 | 82372 ± 2828 |

**Table S14:** Mitochondrial toxicity of HEK293, HSD10, and APP_Swe/Ind_ cells 72 hr post-seeding to galactose media. Values are given as means ± SD from three independent cell culture preparations with four technical replicates (n=12). SD; standard deviation.

| **Cell line** |  | **% of ATP levels** |  | **% of Dead-cell protease activity** |
| --- | --- | --- | --- | --- |
| **HEK293_wt_** |  | 100.00 ± 4.61 |  | 0.00 ± 0.13 |
| **HSD10** |  | 59.20 ± 2.32 |  | 2.61 ± 0.83 |
| **APP_Swe/Ind_** |  | 56.82 ± 2.74 |  | 3.42 ± 0.78 |


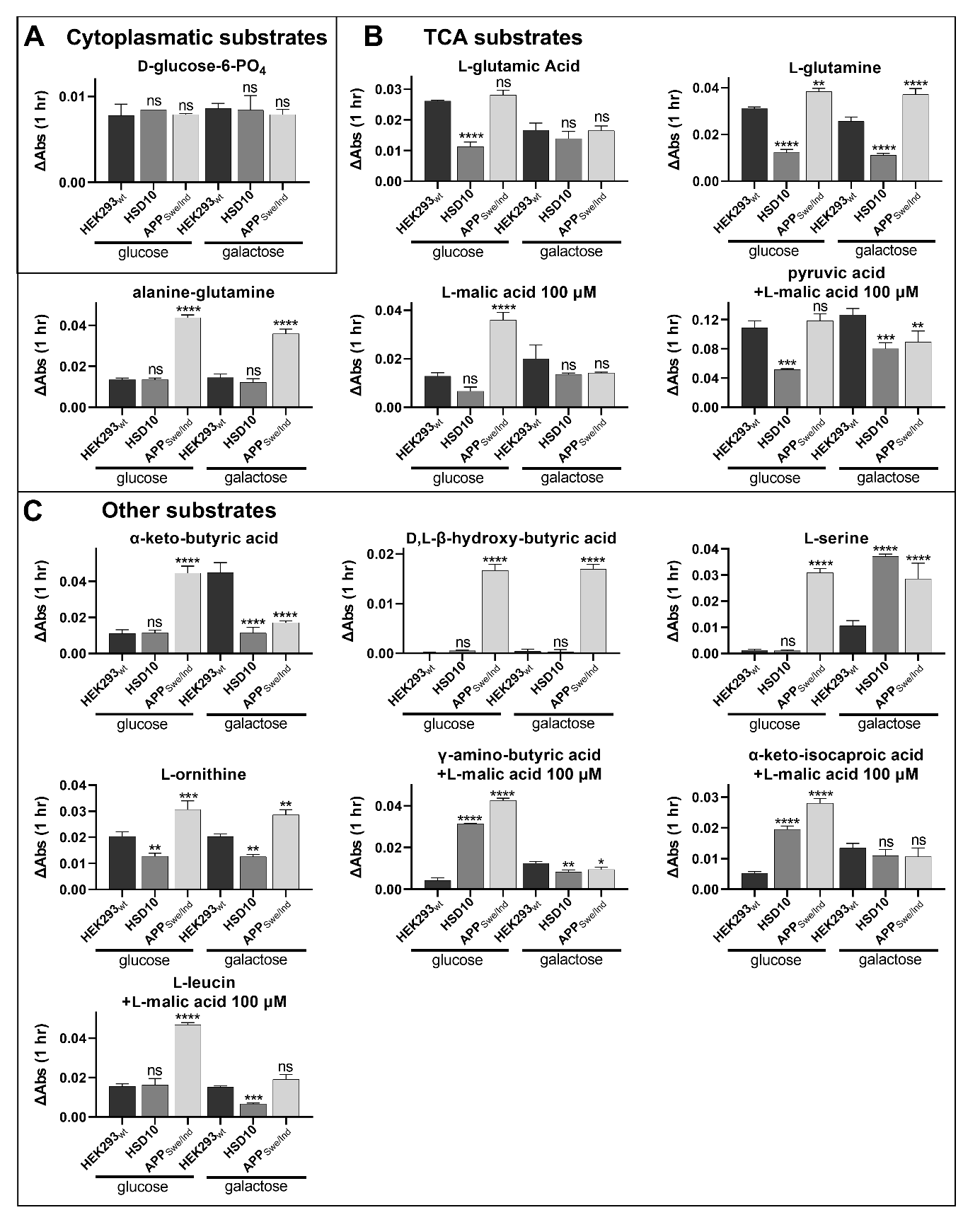


**Figure S15:** The mitochondrial electron flow changes (corresponding to metabolic changes) in HEK293_wt_, HSD10, and APP_Swe/Ind_ cells measured by absorbance (OD_590_) using MitoPlate assay. Conversion of cytosolic substrates (**A**), TCA cycle substrates (**B**), and other substrates (**C**) in HEK293_wt_, HSD10, and APP_Swe/Ind_ cells. Values are given as means ± SD from three independent cell culture preparations with one technical replicate (n=3). SD; standard deviation.

| **Cell line** |  | **Treatment** |  | **% of ATP quantity** |  | **% of Cytotoxicity** |
| --- | --- | --- | --- | --- | --- | --- |
| **HEK293_wt_** |  | untreated |  | 100.00 ± 3.88 |  | 0.00 ± 0.63 |
|  |  | 7.6 nM Aβ42 |  | 77.21 ± 4.23 |  | 13.87 ± 1.92 |
| **HSD10** |  | untreated |  | 74.94 ± 3.04 |  | 18.56 ± 2.82 |
|  |  | 7.6 nM Aβ42 |  | 65.32 ± 3.71 |  | 25.28 ± 3.10 |

**Table S16:** ATP levels and cytotoxicity in HEK293_wt_, and HSD10 cells performed 72 hr post-seeding into the galactose medium containing 7.6 nM Aβ42. Data were normalized between DMSO-treated (1%) and valinomycin-treated (100 µM) HEK293_wt_ cells cultivated in galactose media. Values are given as means ± SD from three independent cell culture preparations with three technical replicates (n=9). SD; standard deviation.

**Table S17:** ATP levels and cytotoxicity in HSD10 cells after HSD10-inhibitors treatment performed 72 hr post-seeding into the galactose medium. Data were normalized between DMSO-treated (1%) and valinomycin-treated (100 µM) HEK293_wt_ cells cultivated in galactose media. Values are given as means ± SD from three independent cell culture preparations with four technical replicates (n=12). SD; standard deviation.

| **Compound** | |  | **% of ATP quantity** |  | **% of Cytotoxicity** |
| --- | --- | --- | --- | --- | --- |
| **DMSO** | 1% |  | 70.69 ± 3.68 |  | 18.08 ± 1.91 |
| **AG18051** | 94 nM |  | 85.94 ± 8.32 |  | 16.05 ± 2.56 |
|  | 187 nM |  | 88.47 ± 7.05 |  | 14.00 ± 1.53 |
|  | 0.38 µM |  | 89.20 ± 6.98 |  | 12.26 ± 1.87 |
|  | 0.57 µM |  | 88.28 ± 5.23 |  | 9.24 ± 1.23 |
|  | 0.75 µM |  | 83.09 ± 6.48 |  | 12.58 ± 1.33 |
| **34** | 2.13 µM |  | 85.47 ± 9.84 |  | 15.31 ± 1.03 |
|  | 4.26 µM |  | 88.96 ± 8.08 |  | 14.63 ± 1.56 |
|  | 8.52 µM |  | 88.42 ± 6.60 |  | 13.38 ± 1.07 |
|  | 12.78 µM |  | 88.64 ± 7.53 |  | 10.26 ± 1.94 |
|  | 17.04 µM |  | 84.94 ± 9.90 |  | 12.29 ± 3.04 |

**Table S18:** ATP levels and cytotoxicity in HSD10 cells performed 72 hr post-seeding into the Aβ42-containing galactose medium and HSD10-inhibitors treatment. Data were normalized between DMSO-treated (1%) and valinomycin-treated (100 µM) HEK293 cells cultivated in galactose media. Values are given as means ± SD from three independent cell culture preparations with three technical replicates (n=9). SD; standard deviation.

| **Compound** |  | **% of ATP quantity** |  | **% of Cytotoxicity** |
| --- | --- | --- | --- | --- |
| **DMSO (1%)**  **Aβ42 (7.6 nM)** |  | 65.32 ± 3.71 |  | 25.28 ± 3.10 |
| **AG18051 (0.57 µM)**  **Aβ42 (7.6 nM)** |  | 65.77 ± 4.96 |  | 24.33 ± 3.72 |
| **34 (12.78 µM)**  **Aβ42 (7.6 nM)** |  | 71.50 ± 6.94 |  | 14.51 ± 3.71 |

**Table S19:** The mitochondrial electron flow measurements (corresponding to metabolic changes) in HEK293_wt_, HSD10, and APP_Swe/Ind_ cells measured by absorbance (OD_590_) using MitoPlate assay performed after 48 hr cultivation in glucose or galactose medium.

| **Cultivation conditions** | | **Glucose** | | | **Galactose** | | |
| --- | --- | --- | --- | --- | --- | --- | --- |
| **Cell line** | | **HEK293_wt_** | **HSD10** | **APP_Swe/Ind_** | **HEK293_wt_** | **HSD10** | **APP_Swe/Ind_** |
| **α-D-glucose** | ΔAbs (1 hr) | 0.0077 | 0.0001 | 0.0188 | 0.0059 | 0.0002 | 0.0181 |
|  | SD | 0.0007 | 0.0002 | 0.0014 | 0.0015 | 0.0003 | 0.0015 |
| **glycogen** | ΔAbs (1 hr) | 0.1356 | 0.1191 | 0.1395 | 0.1224 | 0.1160 | 0.1226 |
|  | SD | 0.0211 | 0.0090 | 0.0175 | 0.0181 | 0.0089 | 0.0065 |
| **D-glucose-1-PO4** | ΔAbs (1 hr) | 0.0140 | 0.0048 | 0.0225 | 0.0121 | 0.0051 | 0.0223 |
|  | SD | 0.0010 | 0.0004 | 0.0033 | 0.0005 | 0.0013 | 0.0018 |
| **D-glucose-6-PO4** | ΔAbs (1 hr) | 0.0078 | 0.0084 | 0.0079 | 0.0086 | 0.0084 | 0.0079 |
|  | SD | 0.0013 | 0.0000 | 0.0001 | 0.0006 | 0.0017 | 0.0006 |
| **D-gluconate-6-PO4** | ΔAbs (1 hr) | 0.0134 | 0.0107 | 0.0188 | 0.0132 | 0.0100 | 0.0182 |
|  | SD | 0.0015 | 0.0014 | 0.0033 | 0.0017 | 0.0006 | 0.0008 |
| **D,L-α-glycerol-PO4** | ΔAbs (1 hr) | 0.0279 | 0.0125 | 0.0273 | 0.0239 | 0.0129 | 0.0248 |
|  | SD | 0.0023 | 0.0008 | 0.0013 | 0.0030 | 0.0029 | 0.0014 |
| **L-lactic acid** | ΔAbs (1 hr) | 0.0172 | 0.0083 | 0.0452 | 0.0167 | 0.0088 | 0.0439 |
|  | SD | 0.0011 | 0.0004 | 0.0018 | 0.0006 | 0.0016 | 0.0034 |
| **pyruvic acid** | ΔAbs (1 hr) | 0.0432 | 0.0231 | 0.0433 | 0.0397 | 0.0211 | 0.0393 |
|  | SD | 0.0039 | 0.0013 | 0.0044 | 0.0029 | 0.0005 | 0.0042 |
| **citric acid** | ΔAbs (1 hr) | 0.0082 | 0.0079 | 0.0085 | 0.0064 | 0.0061 | 0.0059 |
|  | SD | 0.0010 | 0.0006 | 0.0003 | 0.0007 | 0.0010 | 0.0016 |
| **D,L-isocitric acid** | ΔAbs (1 hr) | 0.0532 | 0.0321 | 0.0330 | 0.0420 | 0.0169 | 0.0203 |
|  | SD | 0.0006 | 0.0023 | 0.0037 | 0.0006 | 0.0026 | 0.0018 |
| **cis-aconitic acid** | ΔAbs (1 hr) | 0.0339 | 0.0036 | 0.0319 | 0.0101 | 0.0002 | 0.0095 |
|  | SD | 0.0051 | 0.0008 | 0.0073 | 0.0027 | 0.0002 | 0.0022 |
| **α-keto-glutaric acid** | ΔAbs (1 hr) | 0.0721 | 0.0443 | 0.0732 | 0.0765 | 0.0466 | 0.0670 |
|  | SD | 0.0068 | 0.0029 | 0.0045 | 0.0006 | 0.0020 | 0.0003 |
| **succinic acid** | ΔAbs (1 hr) | 0.1155 | 0.0548 | 0.1082 | 0.1453 | 0.1356 | 0.1128 |
|  | SD | 0.0039 | 0.0011 | 0.0068 | 0.0055 | 0.0181 | 0.0068 |
| **fumaric acid** | ΔAbs (1 hr) | 0.0871 | 0.0420 | 0.0887 | 0.1105 | 0.0935 | 0.1025 |
|  | SD | 0.0155 | 0.0041 | 0.0091 | 0.0087 | 0.0068 | 0.0099 |
| **L-malic acid** | ΔAbs (1 hr) | 0.1359 | 0.0644 | 0.1810 | 0.1717 | 0.1229 | 0.1173 |
|  | SD | 0.0096 | 0.0086 | 0.0003 | 0.0126 | 0.0161 | 0.0185 |
| **L-glutamic Acid** | ΔAbs (1 hr) | 0.0262 | 0.0113 | 0.0281 | 0.0167 | 0.0139 | 0.0165 |
|  | SD | 0.0002 | 0.0015 | 0.0015 | 0.0023 | 0.0023 | 0.0015 |
| **L-glutamine** | ΔAbs (1 hr) | 0.0310 | 0.0123 | 0.0383 | 0.0257 | 0.0112 | 0.0371 |
|  | SD | 0.0008 | 0.0013 | 0.0014 | 0.0018 | 0.0007 | 0.0026 |
| **alanine-glutamine** | ΔAbs (1 hr) | 0.0136 | 0.0136 | 0.0439 | 0.0145 | 0.0123 | 0.0360 |
|  | SD | 0.0007 | 0.0007 | 0.0013 | 0.0018 | 0.0017 | 0.0023 |
| **L-malic acid 100 μM** | ΔAbs (1 hr) | 0.0129 | 0.0067 | 0.0360 | 0.0200 | 0.0136 | 0.0142 |
|  | SD | 0.0013 | 0.0017 | 0.0031 | 0.0057 | 0.0006 | 0.0003 |
| **pyruvic acid+L-malic acid 100 μM** | ΔAbs (1 hr) | 0.1087 | 0.0515 | 0.1184 | 0.1267 | 0.0803 | 0.0891 |
|  | SD | 0.0096 | 0.0014 | 0.0095 | 0.0084 | 0.0081 | 0.0151 |
| **acetyl-L-carnitine+L-malic acid 100 μM** | ΔAbs (1 hr) | 0.0176 | 0.0106 | 0.0303 | 0.0177 | 0.0092 | 0.0179 |
|  | SD | 0.0018 | 0.0019 | 0.0008 | 0.0014 | 0.0006 | 0.0032 |
| **octanoyl-L-carnitine+L-malic acid 100 μM** | ΔAbs (1 hr) | 0.0143 | 0.0107 | 0.0323 | 0.0140 | 0.0019 | 0.0168 |
|  | SD | 0.0005 | 0.0006 | 0.0011 | 0.0018 | 0.0004 | 0.0006 |
| **palmitoyl-D,L-carnitine+L-malic acid 100 μM** | ΔAbs (1 hr) | 0.0157 | 0.0092 | 0.0355 | 0.0150 | 0.0075 | 0.0217 |
|  | SD | 0.0008 | 0.0011 | 0.0030 | 0.0008 | 0.0002 | 0.0006 |
| **α-keto-butyric acid** | ΔAbs (1 hr) | 0.0110 | 0.0114 | 0.0446 | 0.0448 | 0.0114 | 0.0171 |
|  | SD | 0.0022 | 0.0016 | 0.0039 | 0.0057 | 0.0031 | 0.0009 |
| **D,L-β-hydroxy-butyric acid** | ΔAbs (1 hr) | 0.0001 | 0.0005 | 0.0167 | 0.0004 | 0.0003 | 0.0169 |
|  | SD | 0.0002 | 0.0002 | 0.0012 | 0.0005 | 0.0005 | 0.0010 |
| **L-serine** | ΔAbs (1 hr) | 0.0012 | 0.0011 | 0.0309 | 0.0106 | 0.0372 | 0.0286 |
|  | SD | 0.0004 | 0.0002 | 0.0016 | 0.0020 | 0.0008 | 0.0059 |
| **L-ornithine** | ΔAbs (1 hr) | 0.0204 | 0.0128 | 0.0307 | 0.0203 | 0.0126 | 0.0286 |
|  | SD | 0.0017 | 0.0011 | 0.0032 | 0.0010 | 0.0008 | 0.0020 |
| **tryptamine** | ΔAbs (1 hr) | 0.0114 | 0.0195 | 0.0750 | 0.0115 | 0.0092 | 0.0245 |
|  | SD | 0.0010 | 0.0032 | 0.0011 | 0.0012 | 0.0017 | 0.0031 |
| **γ-amino-butyric acid+L-malic acid 100 μM** | ΔAbs (1 hr) | 0.0043 | 0.0314 | 0.0425 | 0.0126 | 0.0083 | 0.0095 |
|  | SD | 0.0012 | 0.0002 | 0.0012 | 0.0008 | 0.0010 | 0.0011 |
| **α-keto-isocaproic acid+L-malic acid 100 μM** | ΔAbs (1 hr) | 0.0053 | 0.0195 | 0.0281 | 0.0135 | 0.0110 | 0.0107 |
|  | SD | 0.0005 | 0.0011 | 0.0014 | 0.0014 | 0.0020 | 0.0028 |
| **L-leucin+L-malic acid 100 μM** | ΔAbs (1 hr) | 0.0155 | 0.0163 | 0.0468 | 0.0152 | 0.0066 | 0.0191 |
|  | SD | 0.0013 | 0.0032 | 0.0011 | 0.0006 | 0.0005 | 0.0024 |

**
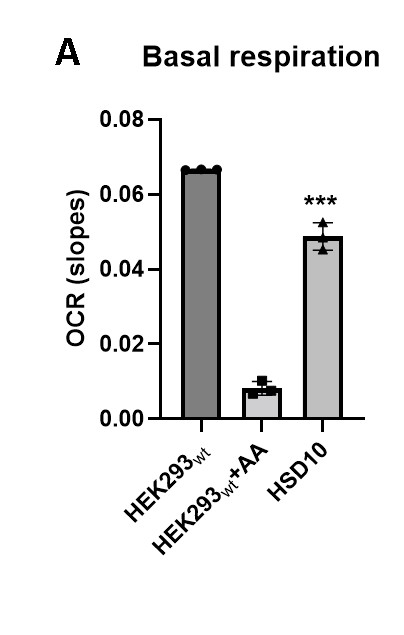
Figure S20:** Oxygen consumption rates in HEK293_wt_ and HSD10 cells. (A) Basal respiration (rates of oxygen consumption) in HEK293_wt_ alone or treated by 1µM antimycin-A, and HSD10 cells. Statistical difference: *p ≤ 0.05, **p ≤ 0.01, ***p ≤ 0.001, compared to the HEK293_wt_ control group.

**
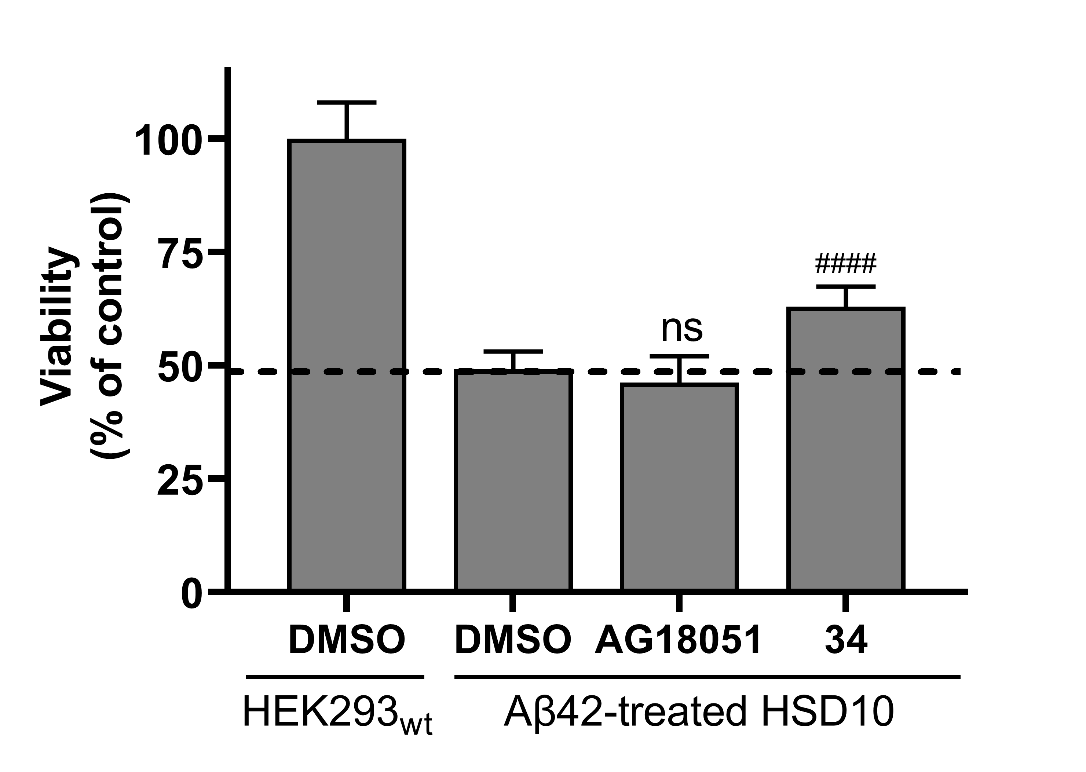
Figure S21:** Viability measurement (using the resazurin) in HSD10 cells after the co-treatment of Aβ42 and inhibitors. Viability in HSD10 cells was measured 72 hr post-seeding into the Aβ42-containing galactose medium with HSD10 inhibitor co-treatment (three times the IC_50_ value; 0.57 µM for **AG18051** or 12.78 µM for inhibitor **34**), using resazurin-based fluorescence detection. Viability was assessed via the reduction of resazurin to fluorescent resorufin. Data were normalized between DMSO-treated (1%) and valinomycin-treated (100 µM) HEK293_wt_ cells cultivated in galactose medium. Values are given as means ± SD from three independent cell culture preparations with three technical replicates (n=9).
